# Supplementary material for: Caring for Patients with Opioid Use Disorder: A Near-Peer Workshop for Medical Students
Source: MedEdPORTAL. 2026 Jan 27;22:11573. doi: 10.15766/mep_2374-8265.11573 (PMC12835442; doi:10.15766/mep_2374-8265.11573)
Supplement: Supplementary file 1 — Presentation Materials.pptxPostsession Handout.docxFacilitator Guide.docxSurveys.docx [file mep_2374-8265.11573-s001.zip › A. Presentation Materials.pptx]

## Slide 1
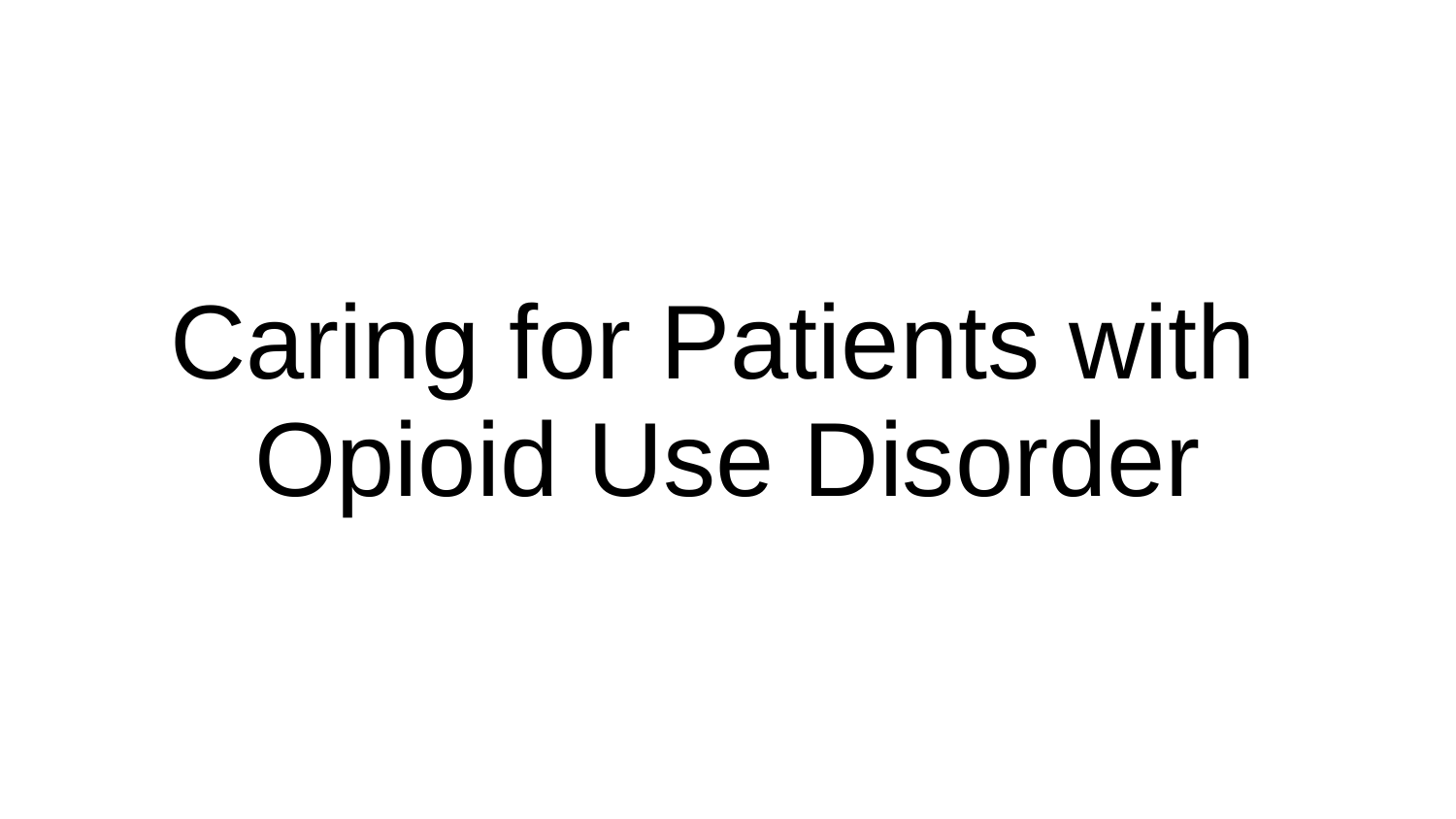

# Caring for Patients with
Opioid Use Disorder

## Slide 2
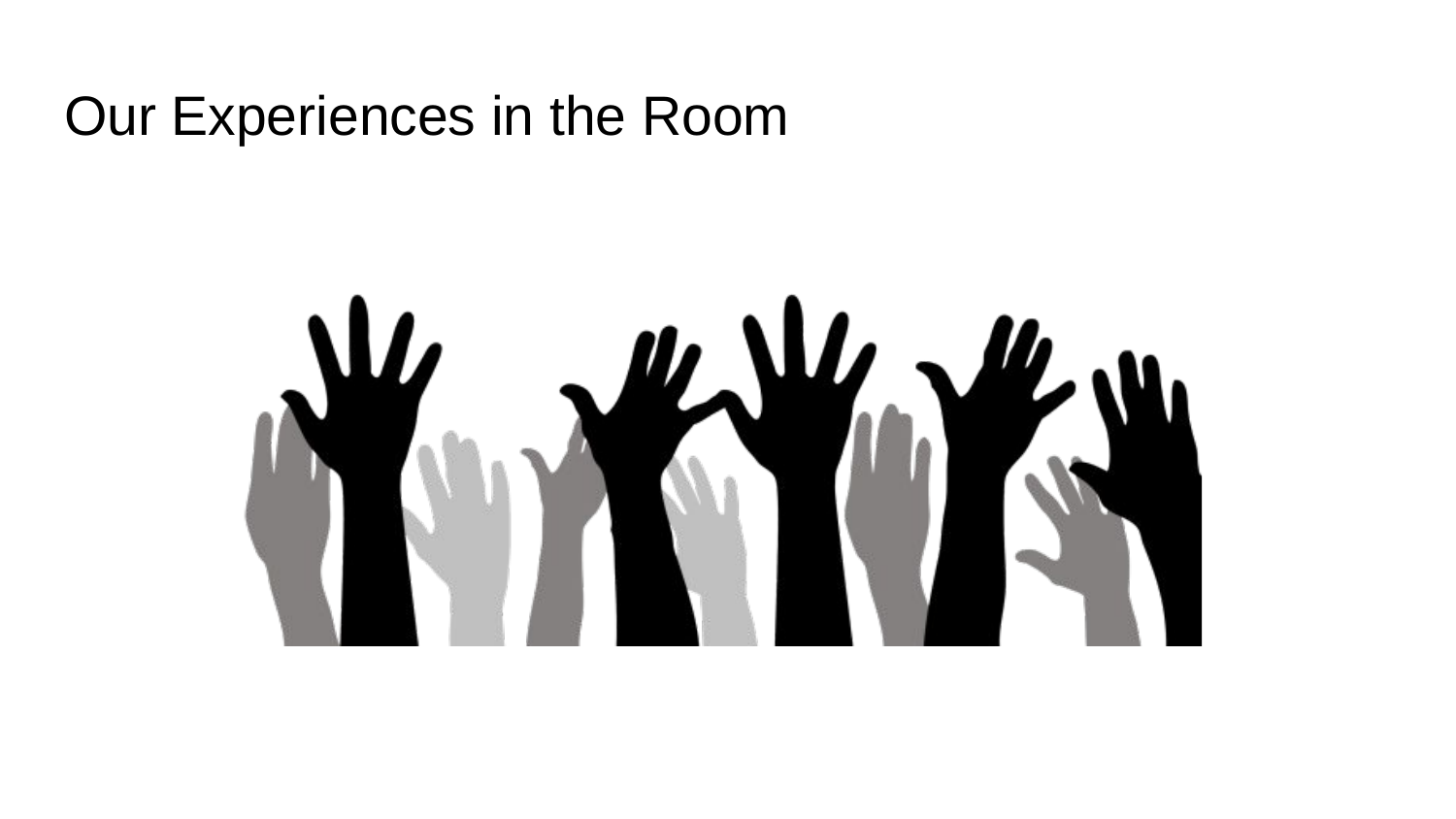

# Our Experiences in the Room

## Slide 3
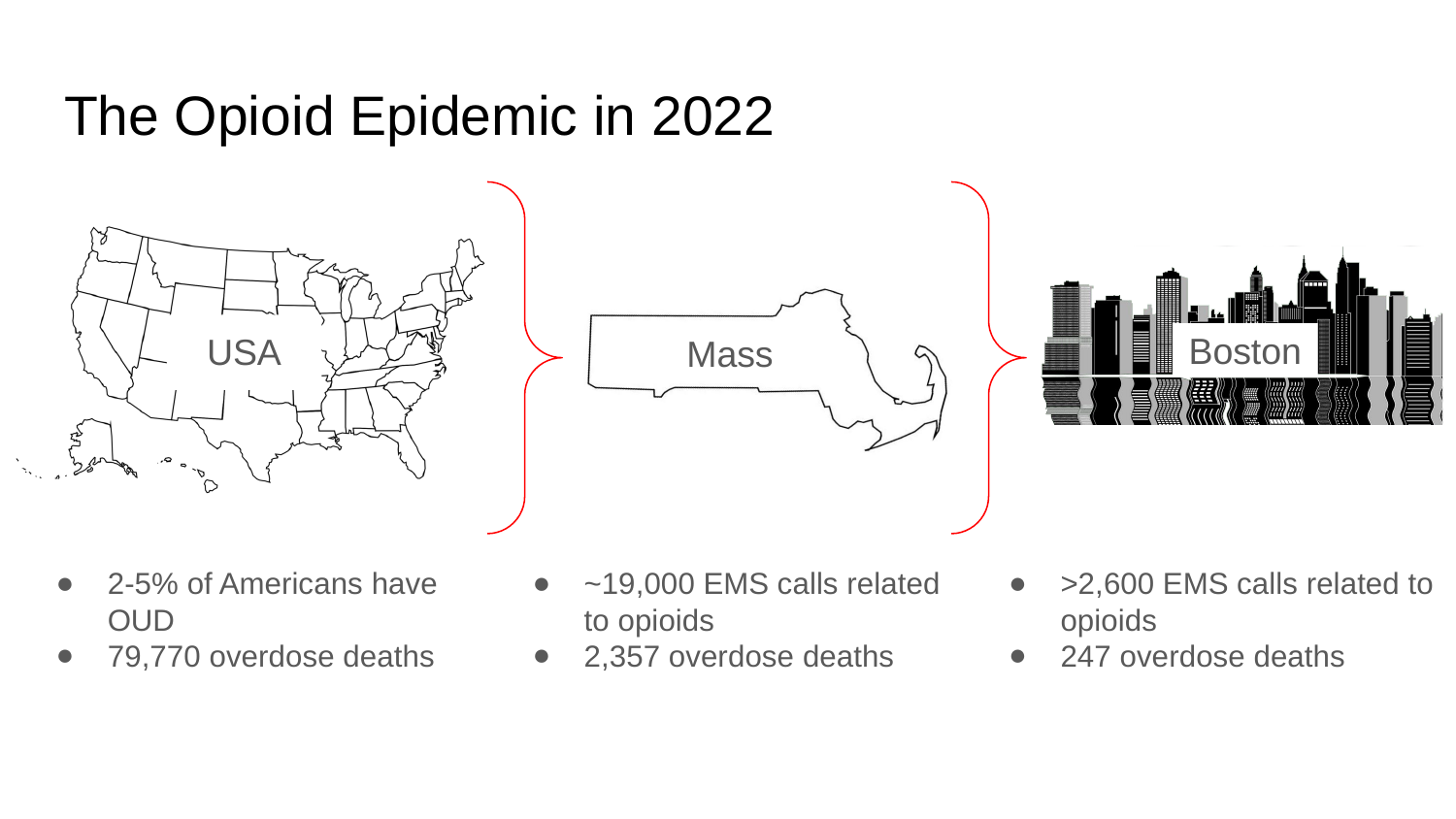

# The Opioid Epidemic in 2022
USA
Mass
Boston
2-5% of Americans have OUD
79,770 overdose deaths
~19,000 EMS calls related to opioids
2,357 overdose deaths
>2,600 EMS calls related to opioids
247 overdose deaths

## Slide 4
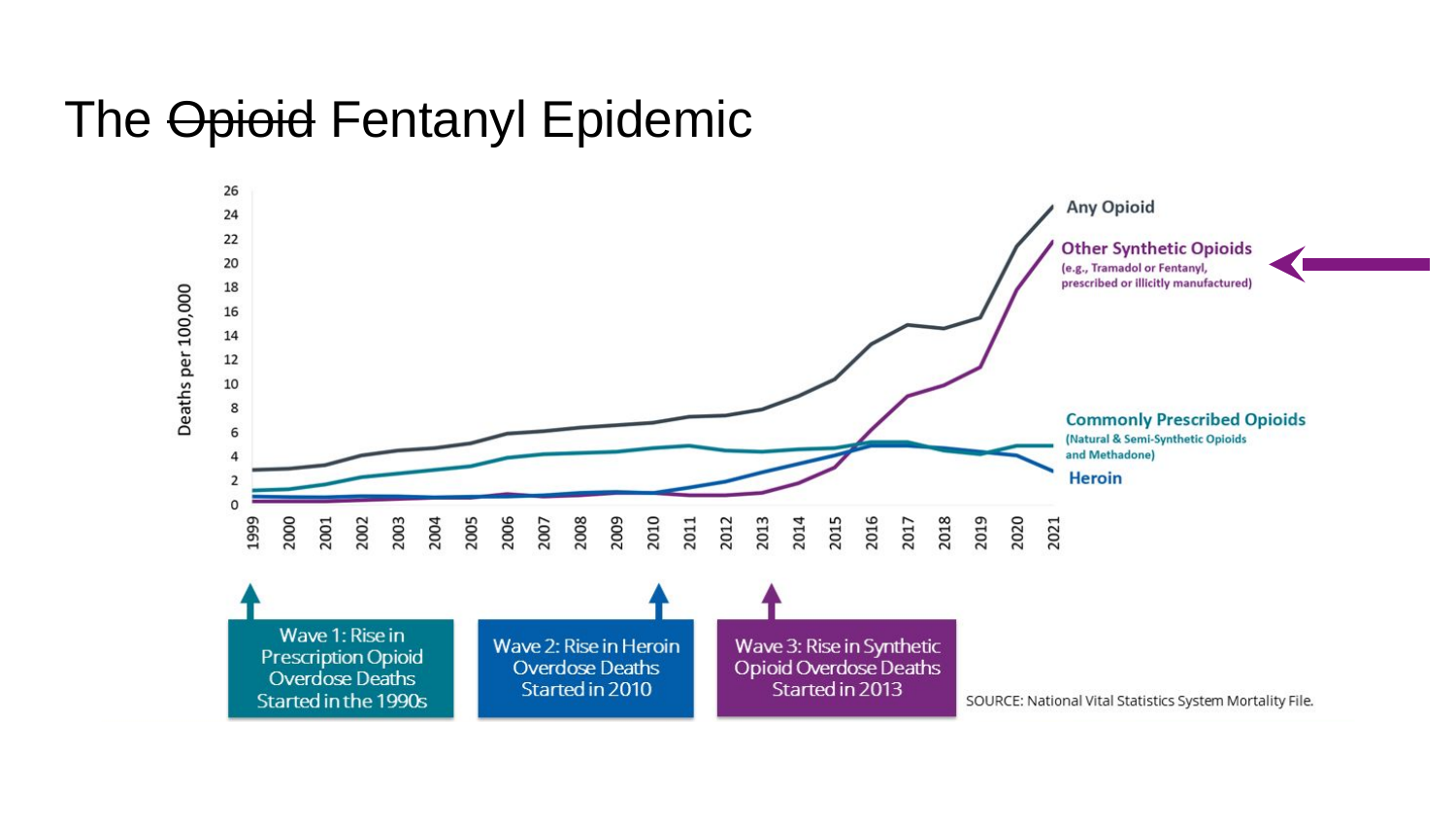

# The Opioid Fentanyl Epidemic

## Slide 5
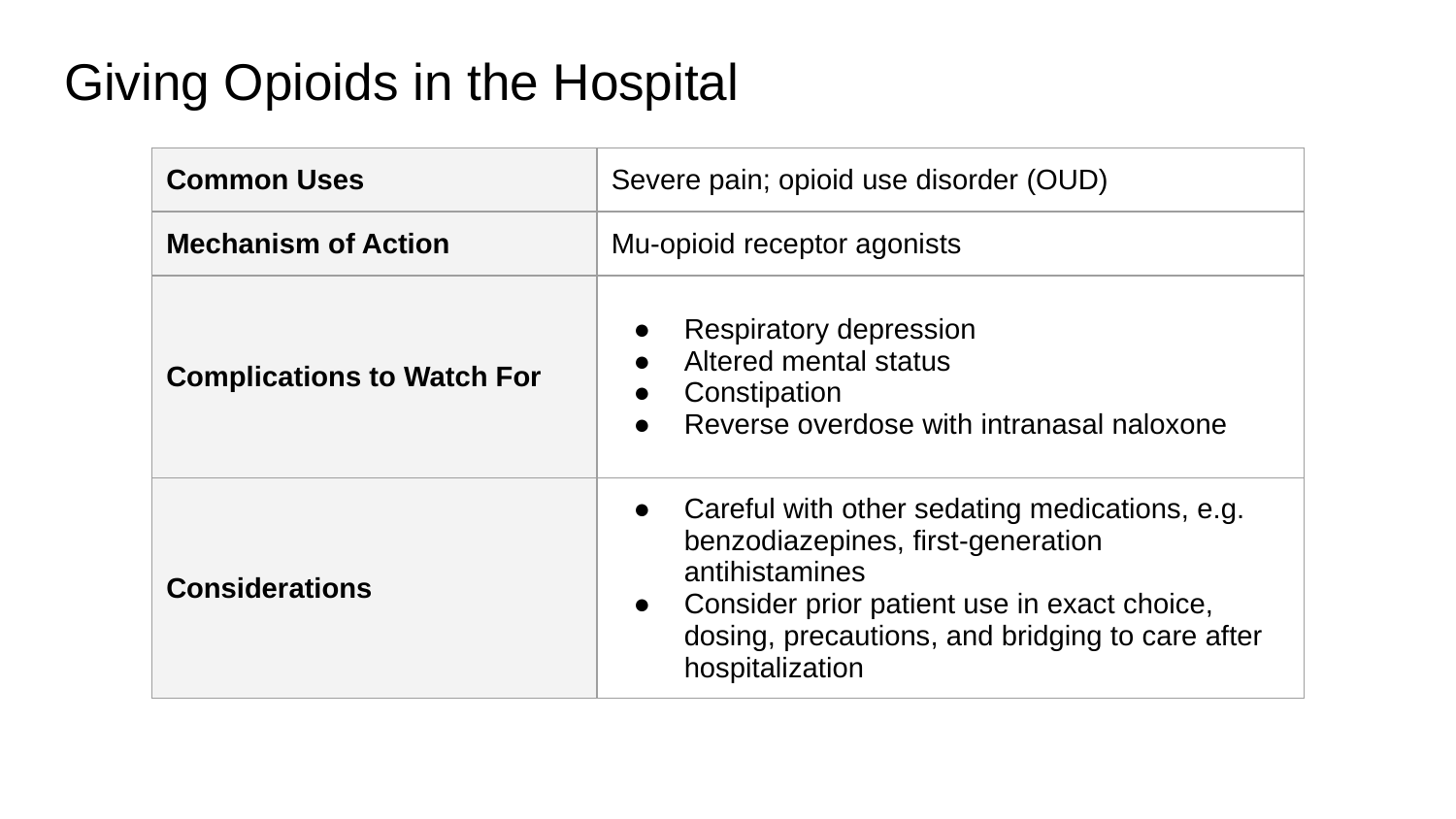

# Giving Opioids in the Hospital
| Common Uses | Severe pain; opioid use disorder (OUD) |
| --- | --- |
| Mechanism of Action | Mu-opioid receptor agonists |
| Complications to Watch For | Respiratory depression Altered mental status Constipation Reverse overdose with intranasal naloxone |
| Considerations | Careful with other sedating medications, e.g. benzodiazepines, first-generation antihistamines Consider prior patient use in exact choice, dosing, precautions, and bridging to care after hospitalization |

## Slide 6
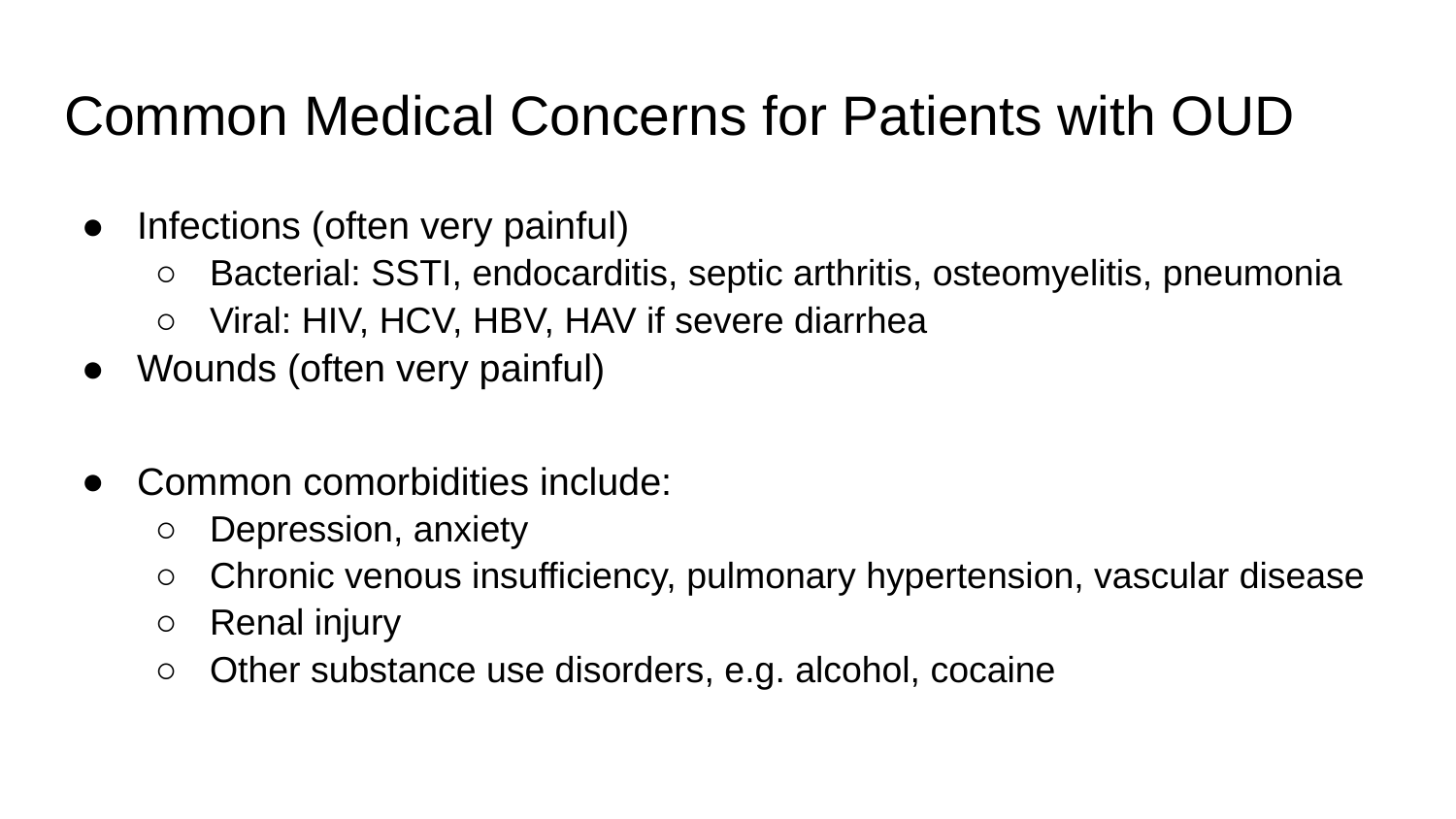

# Common Medical Concerns for Patients with OUD
Infections (often very painful)
Bacterial: SSTI, endocarditis, septic arthritis, osteomyelitis, pneumonia
Viral: HIV, HCV, HBV, HAV if severe diarrhea
Wounds (often very painful)
Common comorbidities include:
Depression, anxiety
Chronic venous insufficiency, pulmonary hypertension, vascular disease
Renal injury
Other substance use disorders, e.g. alcohol, cocaine

## Slide 7
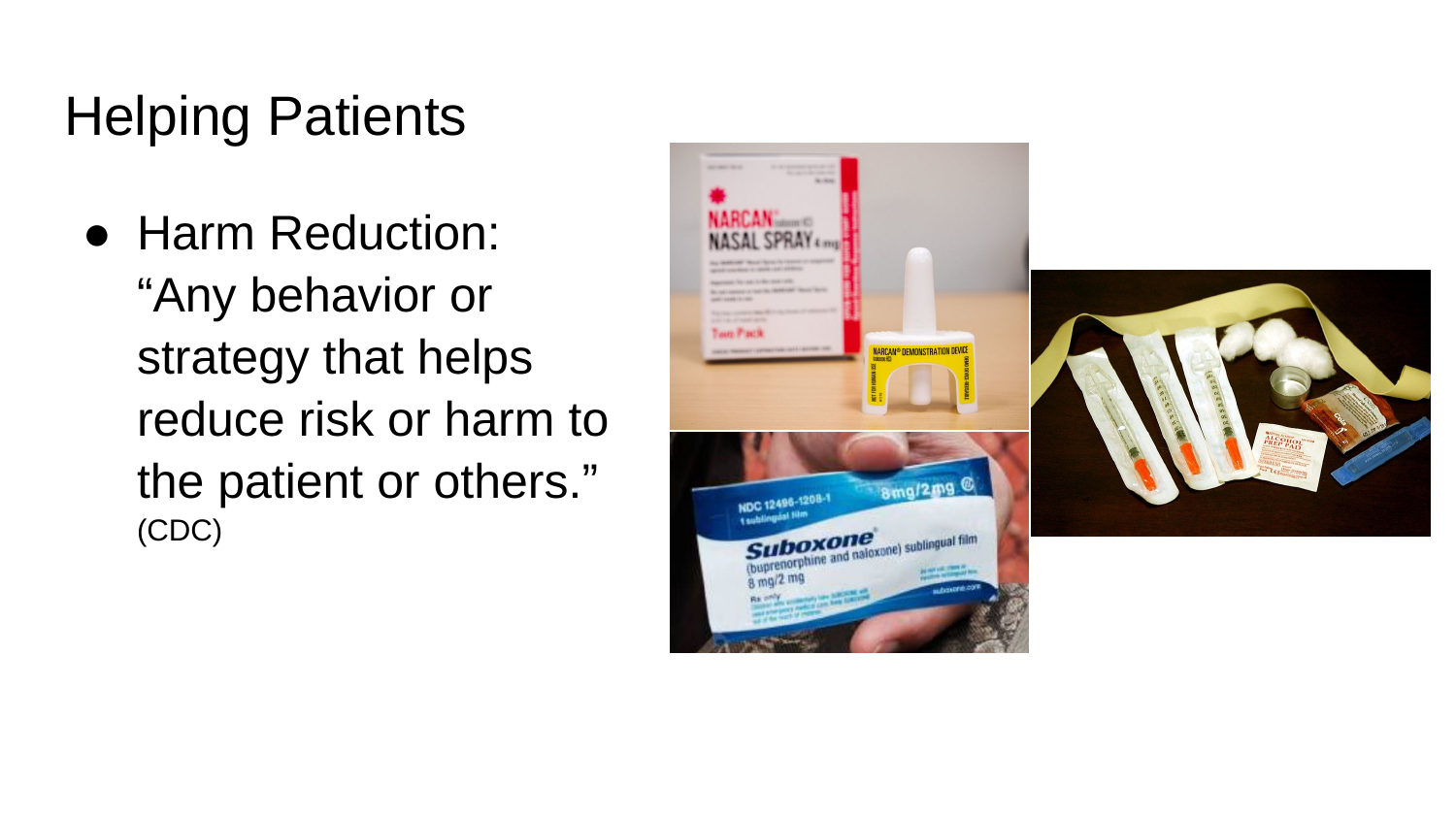

# Helping Patients
Harm Reduction: “Any behavior or strategy that helps reduce risk or harm to the patient or others.” (CDC)

## Slide 8
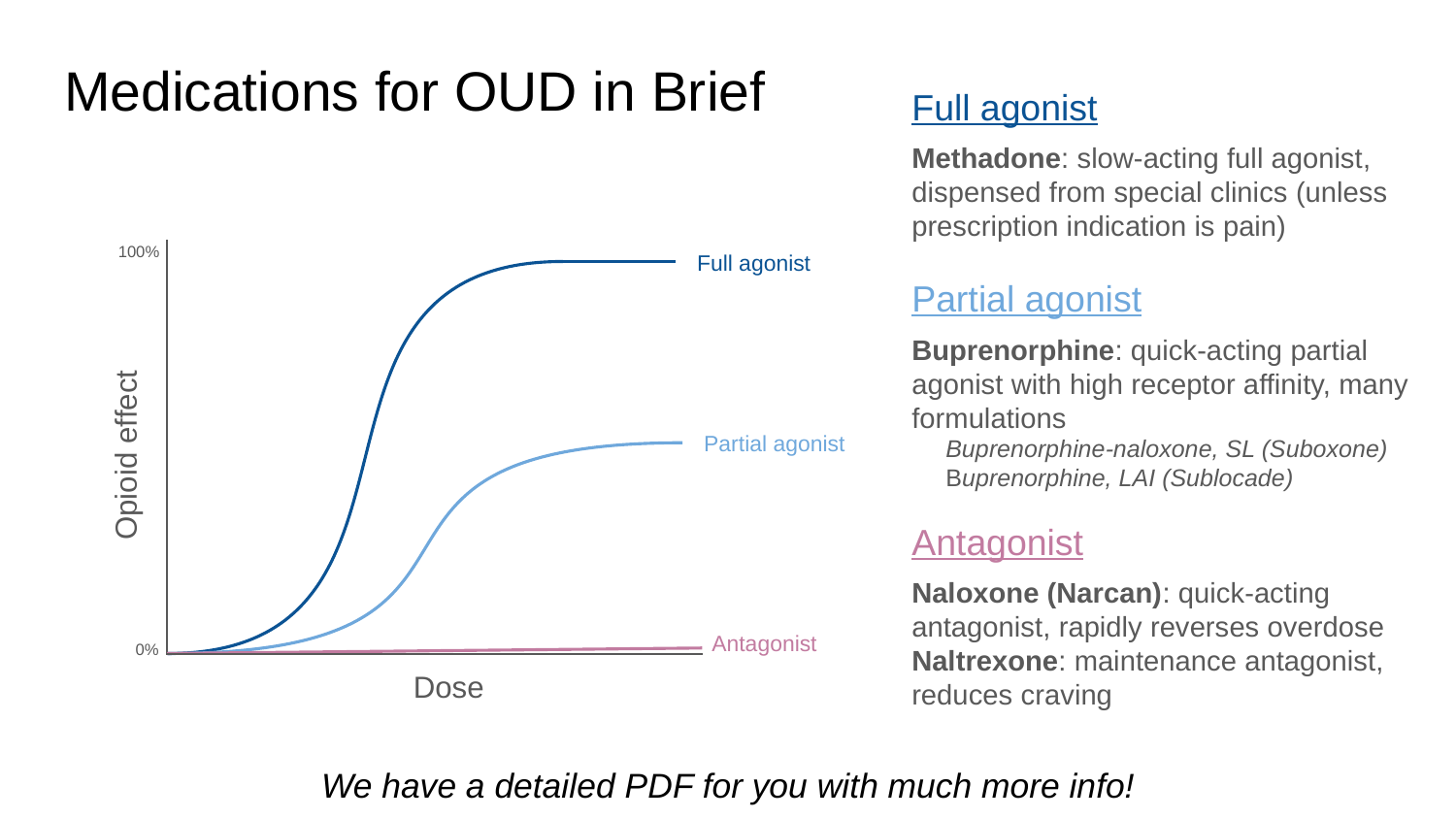

# Medications for OUD in Brief
Full agonist
Methadone: slow-acting full agonist, dispensed from special clinics (unless prescription indication is pain)
100%
Full agonist
Partial agonist
Buprenorphine: quick-acting partial agonist with high receptor affinity, many formulations
 Buprenorphine-naloxone, SL (Suboxone)
 Buprenorphine, LAI (Sublocade)
Partial agonist
Opioid effect
Antagonist
Naloxone (Narcan): quick-acting antagonist, rapidly reverses overdose
Naltrexone: maintenance antagonist, reduces craving
Antagonist
0%
Dose
We have a detailed PDF for you with much more info!

## Slide 9
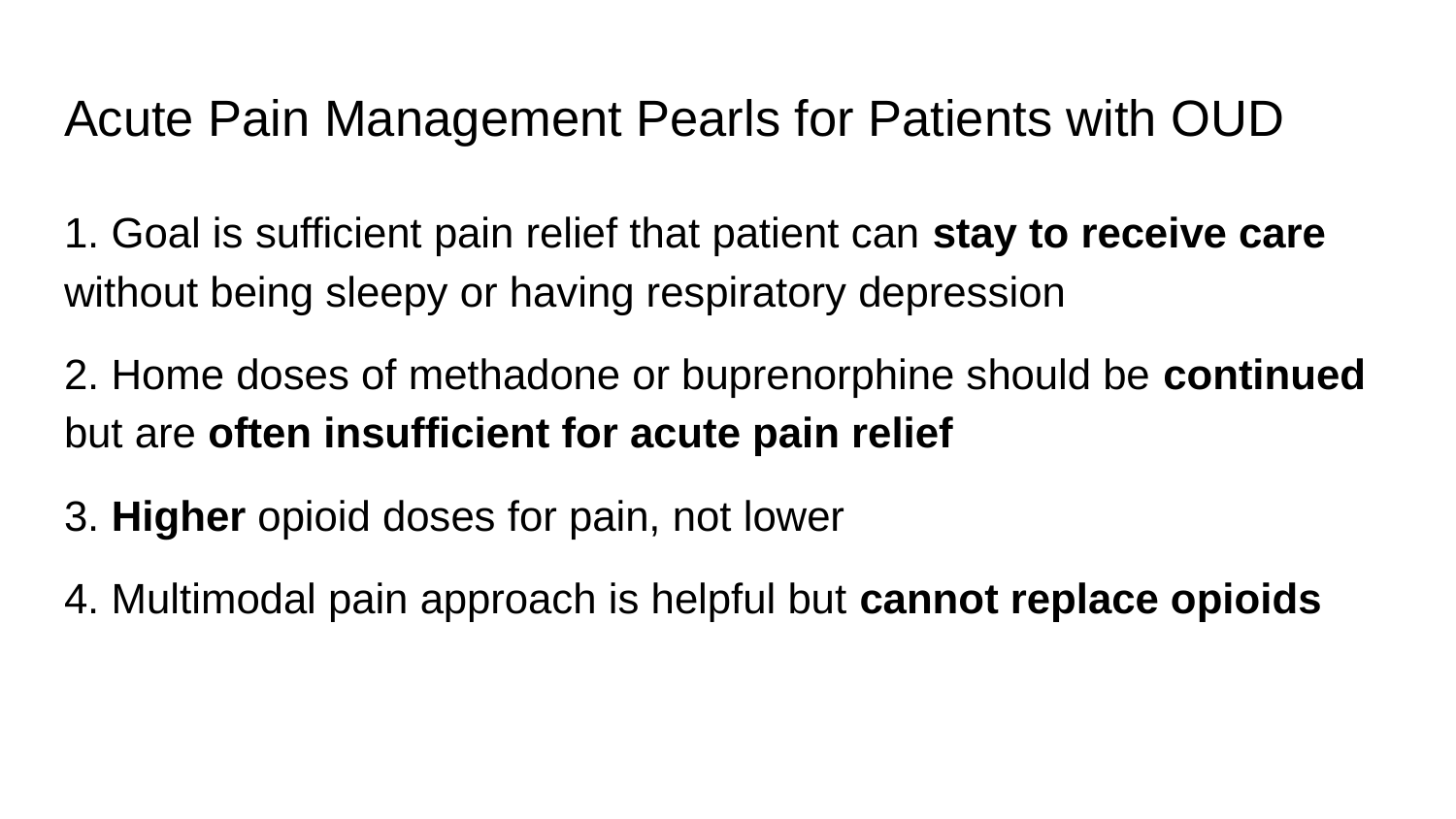

# Acute Pain Management Pearls for Patients with OUD
1. Goal is sufficient pain relief that patient can stay to receive care without being sleepy or having respiratory depression
2. Home doses of methadone or buprenorphine should be continued but are often insufficient for acute pain relief
3. Higher opioid doses for pain, not lower
4. Multimodal pain approach is helpful but cannot replace opioids

## Slide 10
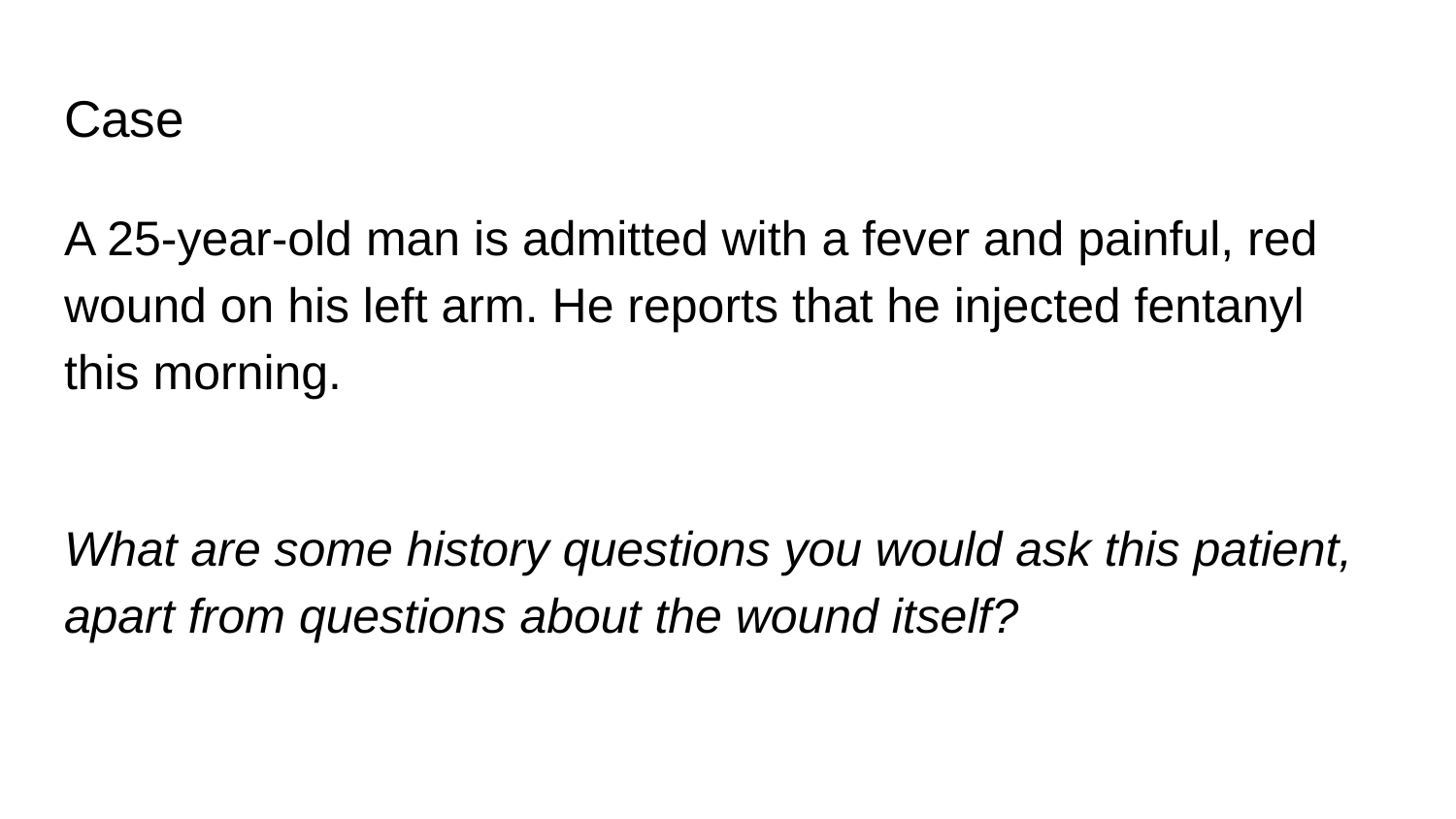

# Case
A 25-year-old man is admitted with a fever and painful, red wound on his left arm. He reports that he injected fentanyl this morning.
What are some history questions you would ask this patient, apart from questions about the wound itself?

## Slide 11
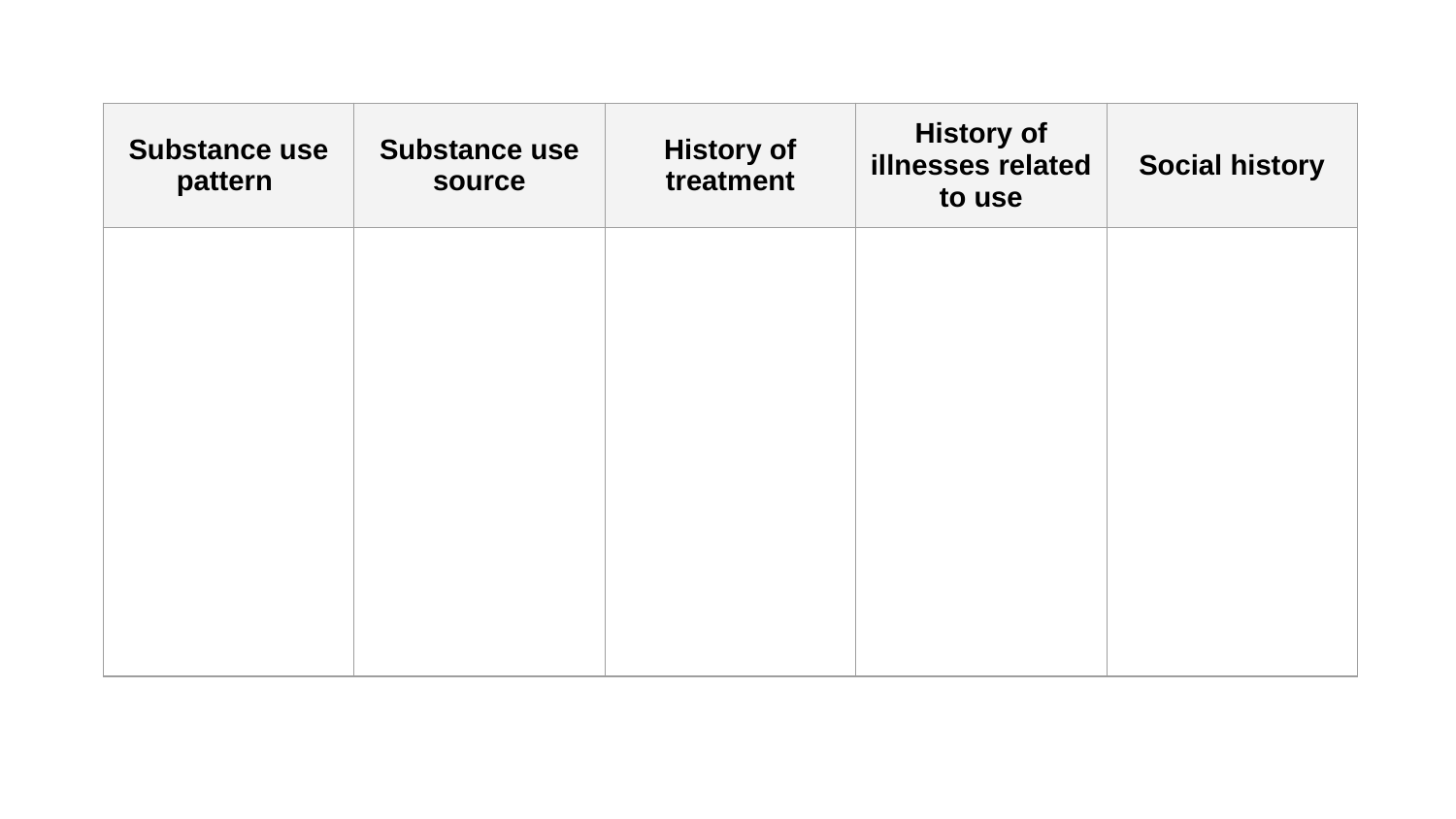

| Substance use pattern | Substance use source | History of treatment | History of illnesses related to use | Social history |
| --- | --- | --- | --- | --- |
| | | | | |

## Slide 12
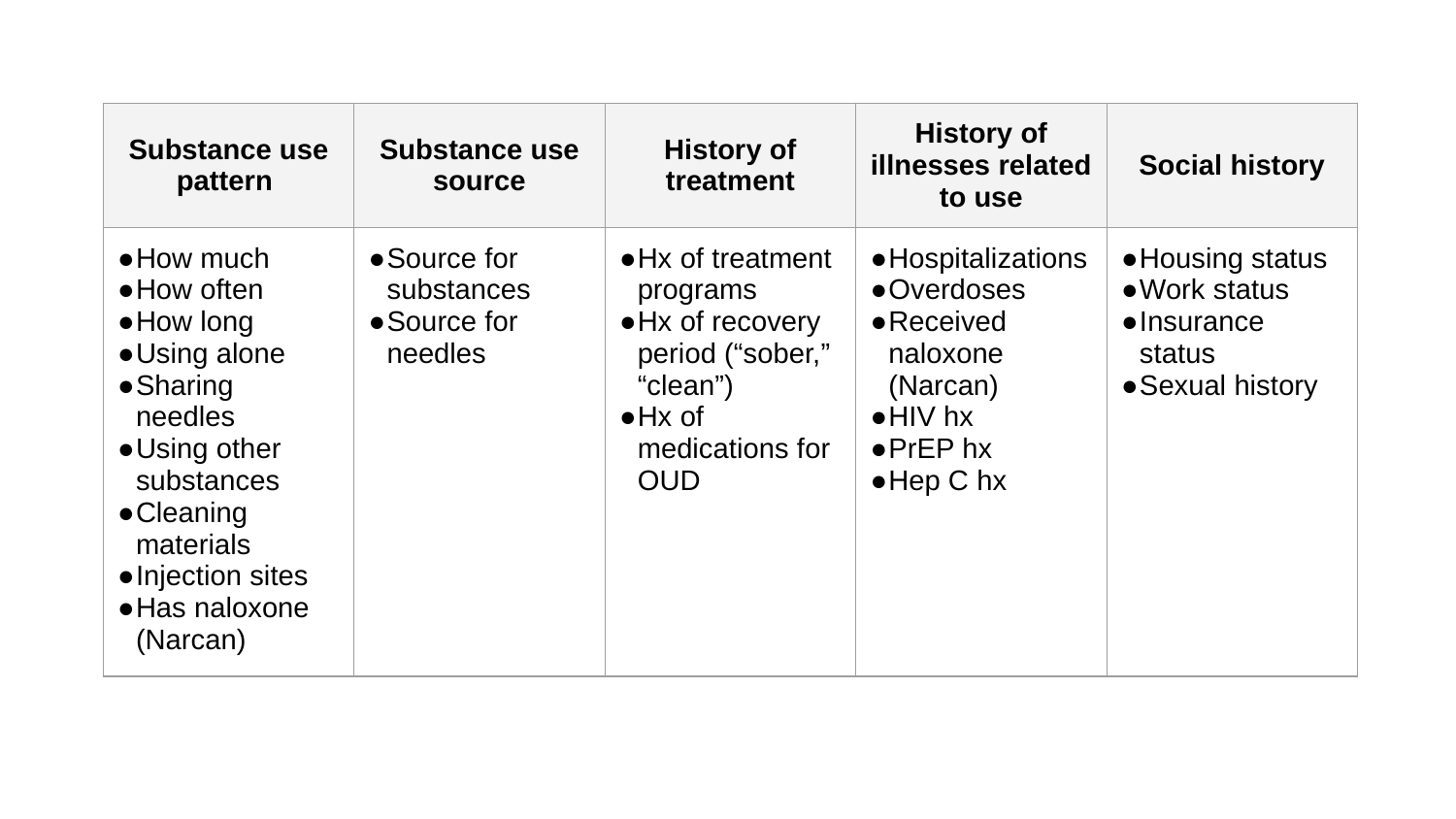

| Substance use pattern | Substance use source | History of treatment | History of illnesses related to use | Social history |
| --- | --- | --- | --- | --- |
| How much How often How long Using alone Sharing needles Using other substances Cleaning materials Injection sites Has naloxone (Narcan) | Source for substances Source for needles | Hx of treatment programs Hx of recovery period (“sober,” “clean”) Hx of medications for OUD | Hospitalizations Overdoses Received naloxone (Narcan) HIV hx PrEP hx Hep C hx | Housing status Work status Insurance status Sexual history |

## Slide 13
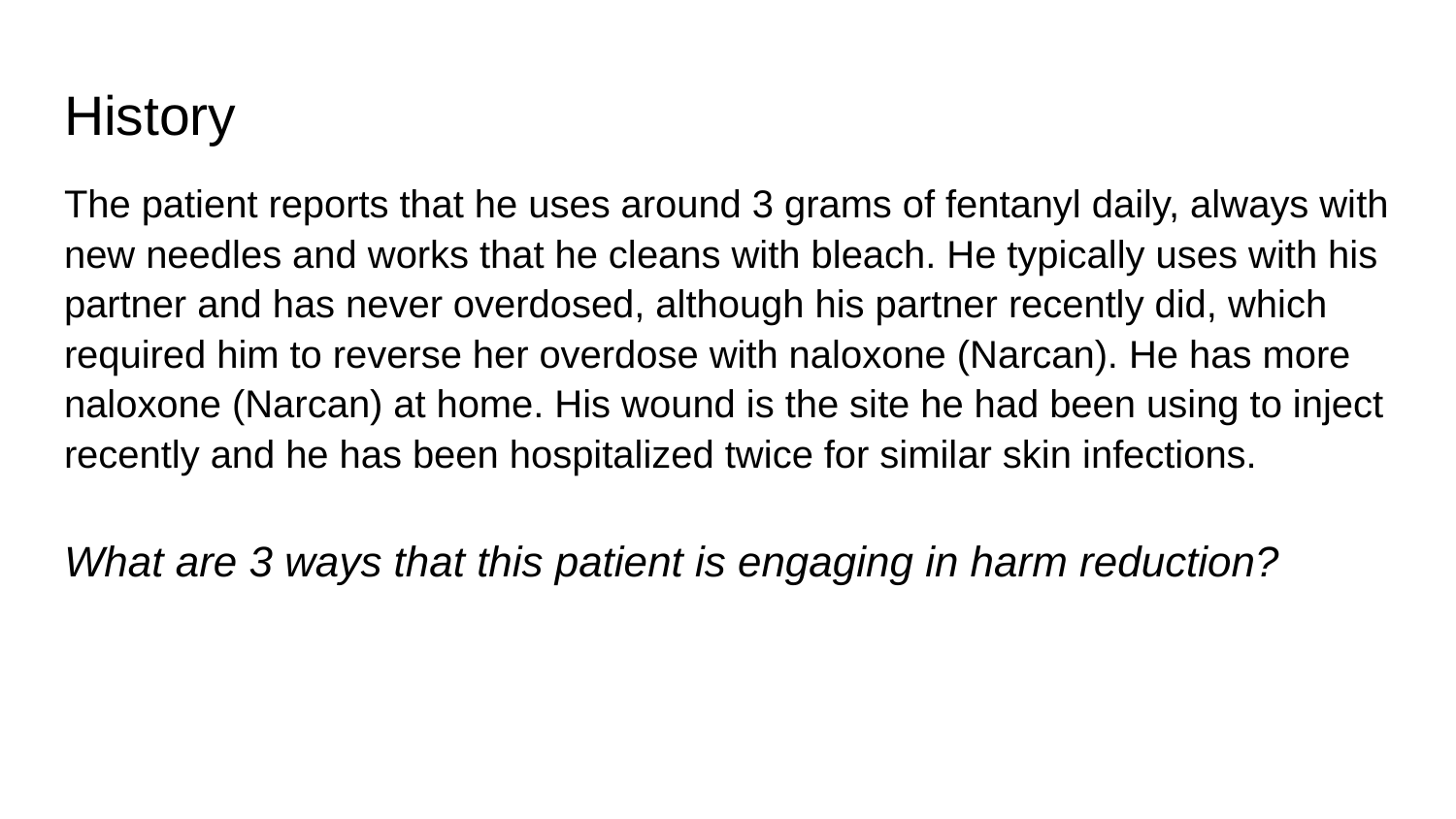

# History
The patient reports that he uses around 3 grams of fentanyl daily, always with new needles and works that he cleans with bleach. He typically uses with his partner and has never overdosed, although his partner recently did, which required him to reverse her overdose with naloxone (Narcan). He has more naloxone (Narcan) at home. His wound is the site he had been using to inject recently and he has been hospitalized twice for similar skin infections.
What are 3 ways that this patient is engaging in harm reduction?

## Slide 14
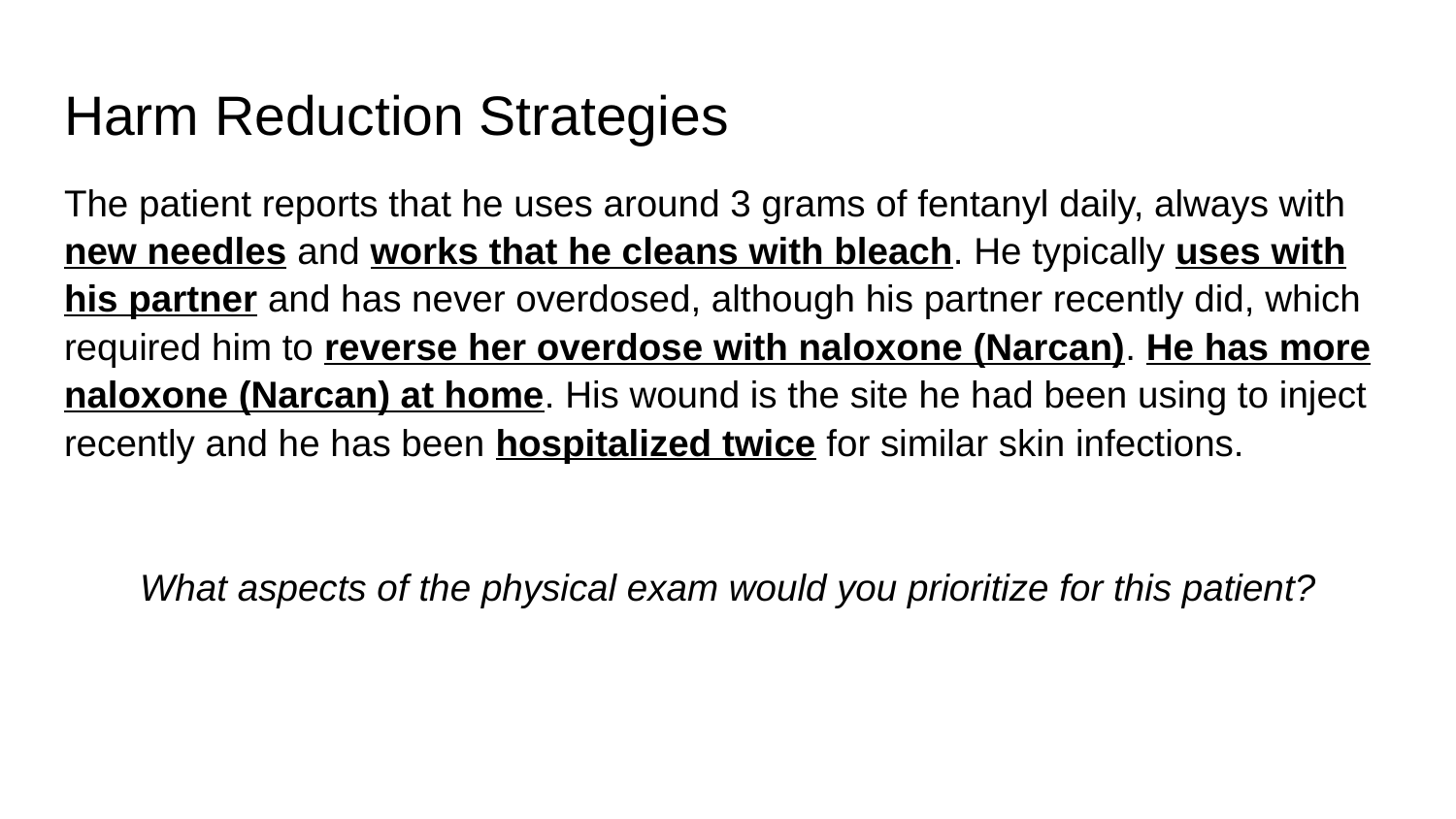

# Harm Reduction Strategies
The patient reports that he uses around 3 grams of fentanyl daily, always with new needles and works that he cleans with bleach. He typically uses with his partner and has never overdosed, although his partner recently did, which required him to reverse her overdose with naloxone (Narcan). He has more naloxone (Narcan) at home. His wound is the site he had been using to inject recently and he has been hospitalized twice for similar skin infections.
What aspects of the physical exam would you prioritize for this patient?

## Slide 15
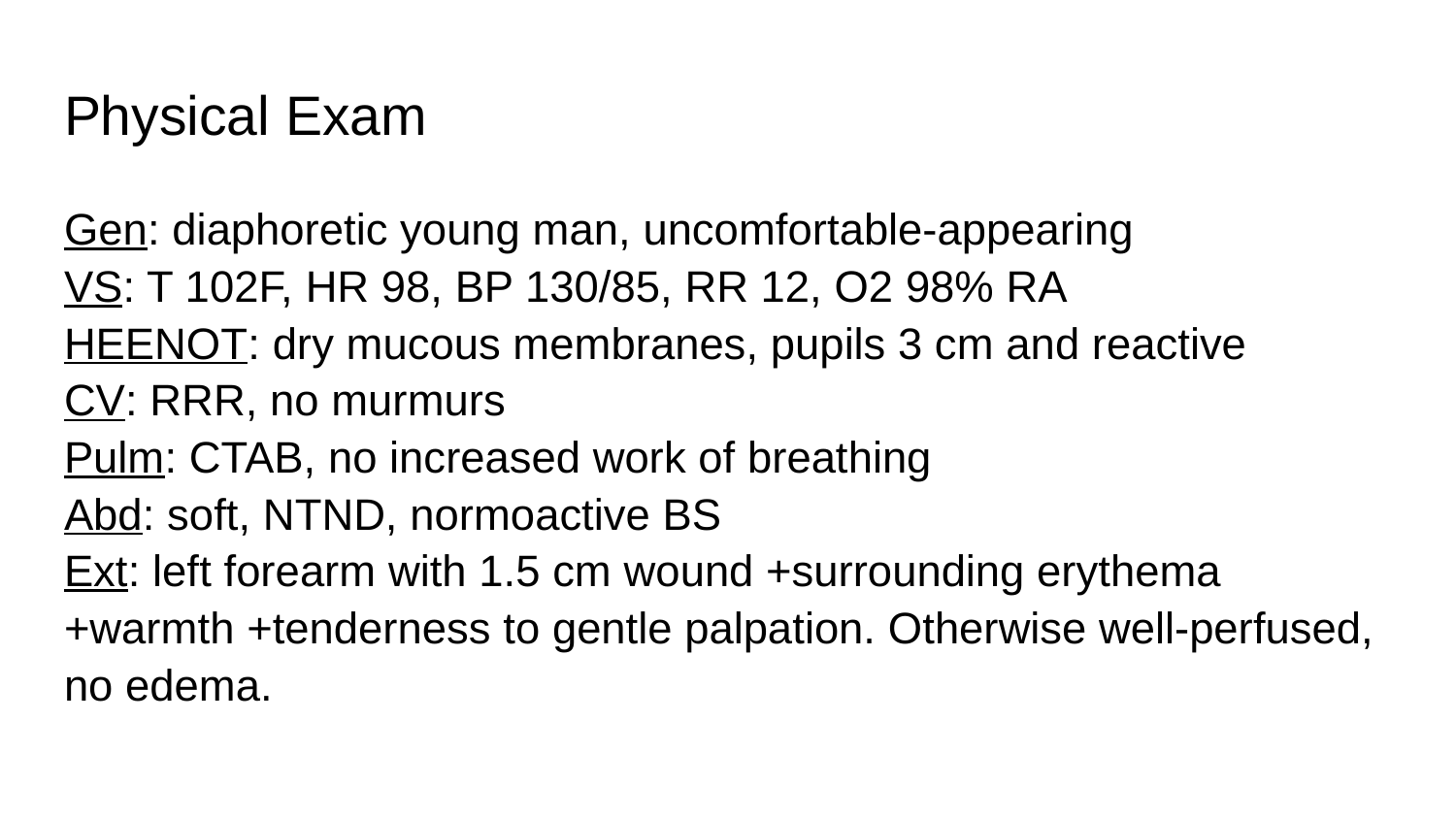

# Physical Exam
Gen: diaphoretic young man, uncomfortable-appearing
VS: T 102F, HR 98, BP 130/85, RR 12, O2 98% RA
HEENOT: dry mucous membranes, pupils 3 cm and reactive
CV: RRR, no murmurs
Pulm: CTAB, no increased work of breathing
Abd: soft, NTND, normoactive BS
Ext: left forearm with 1.5 cm wound +surrounding erythema +warmth +tenderness to gentle palpation. Otherwise well-perfused, no edema.

## Slide 16
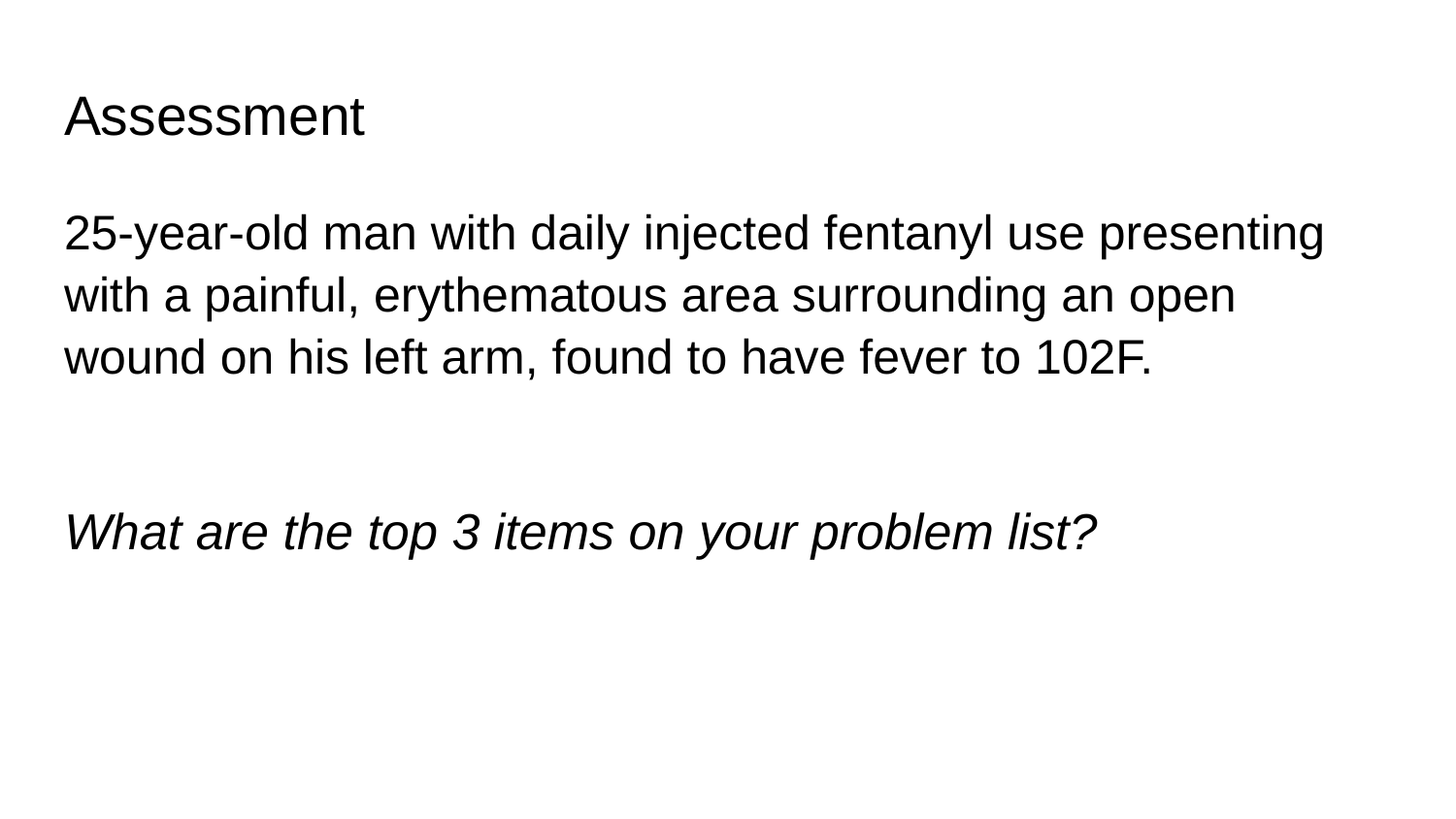

# Assessment
25-year-old man with daily injected fentanyl use presenting with a painful, erythematous area surrounding an open wound on his left arm, found to have fever to 102F.
What are the top 3 items on your problem list?

## Slide 17
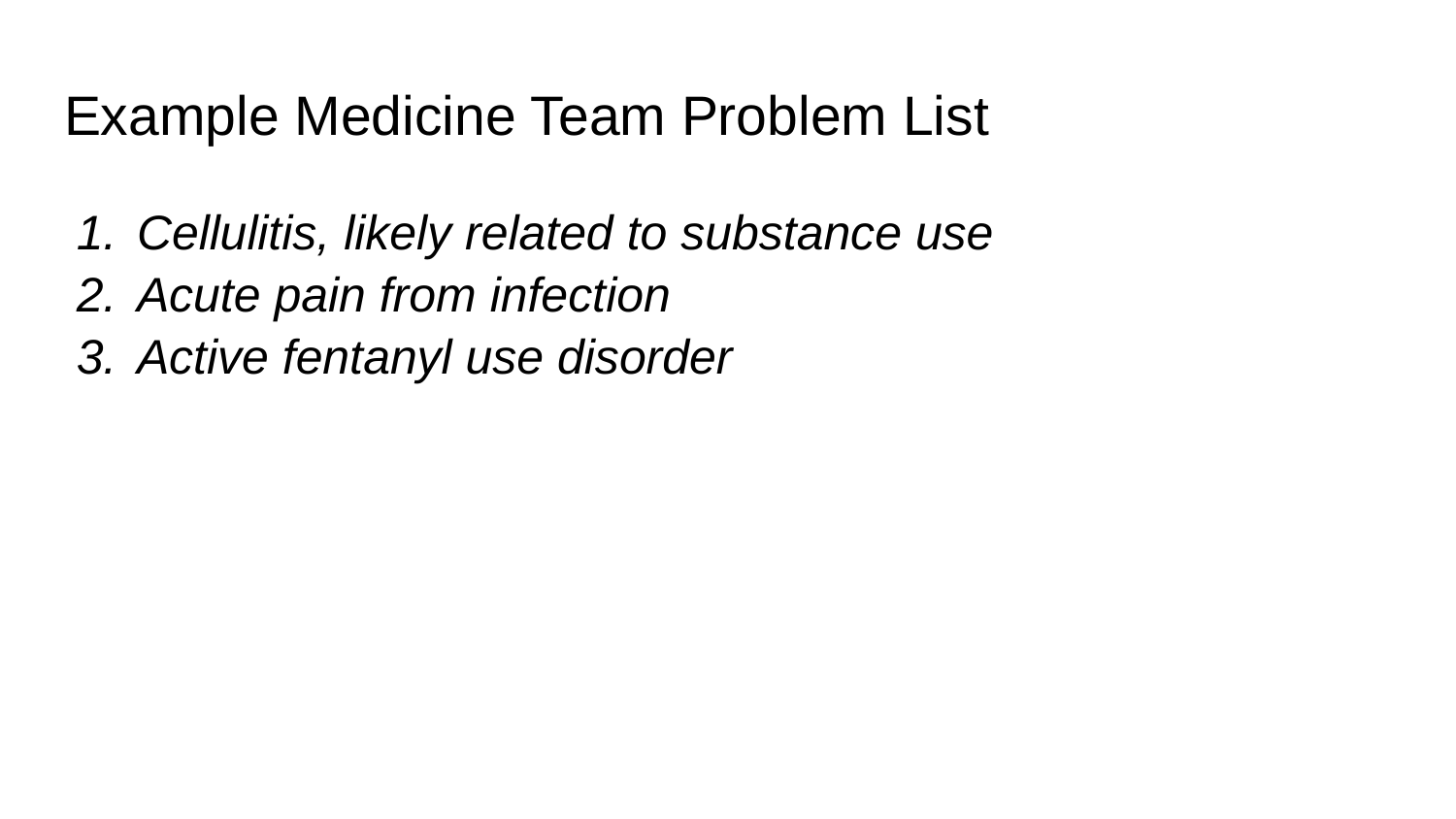

# Example Medicine Team Problem List
Cellulitis, likely related to substance use
Acute pain from infection
Active fentanyl use disorder

## Slide 18
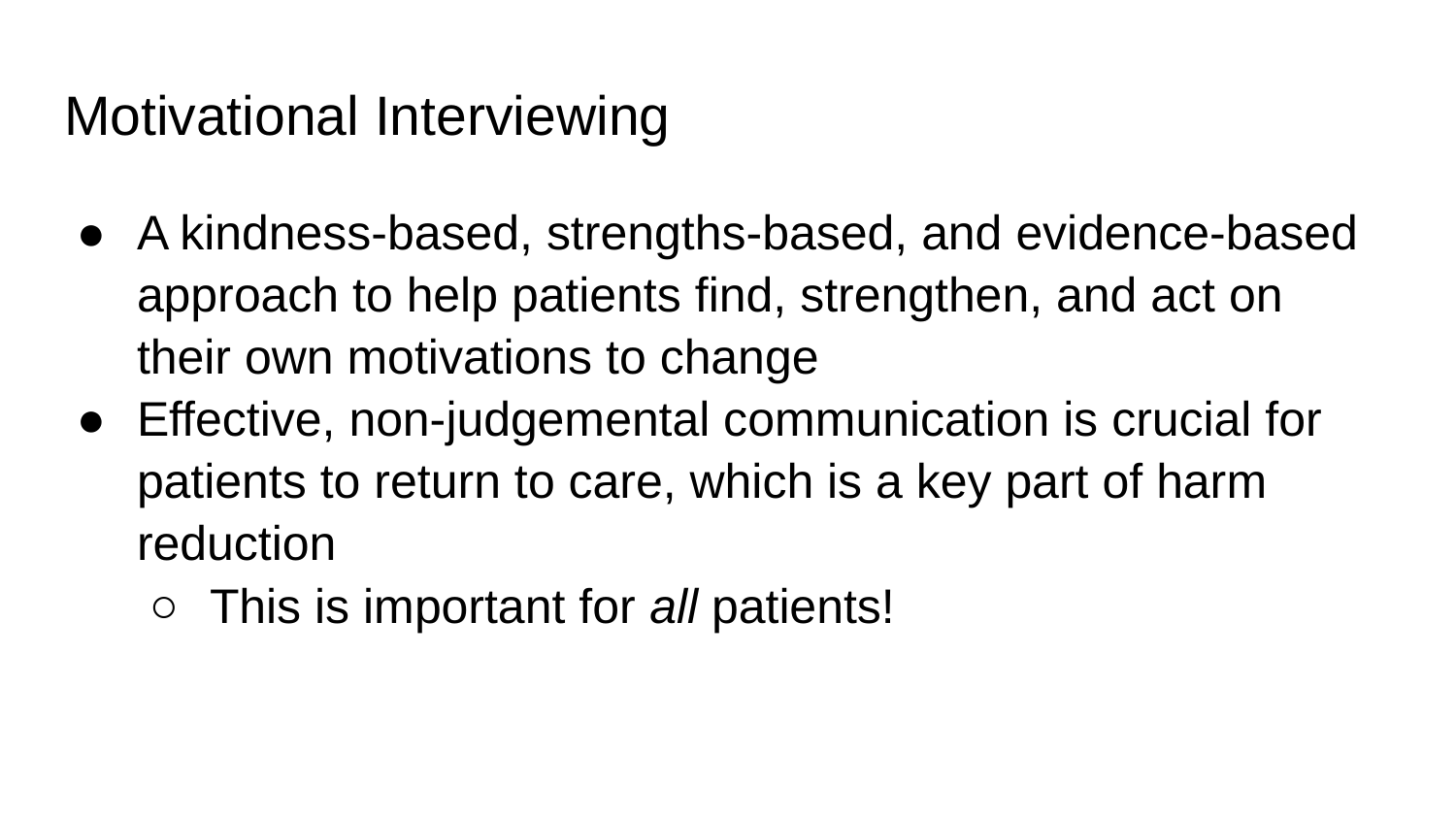

# Motivational Interviewing
A kindness-based, strengths-based, and evidence-based approach to help patients find, strengthen, and act on their own motivations to change
Effective, non-judgemental communication is crucial for patients to return to care, which is a key part of harm reduction
This is important for all patients!

## Slide 19
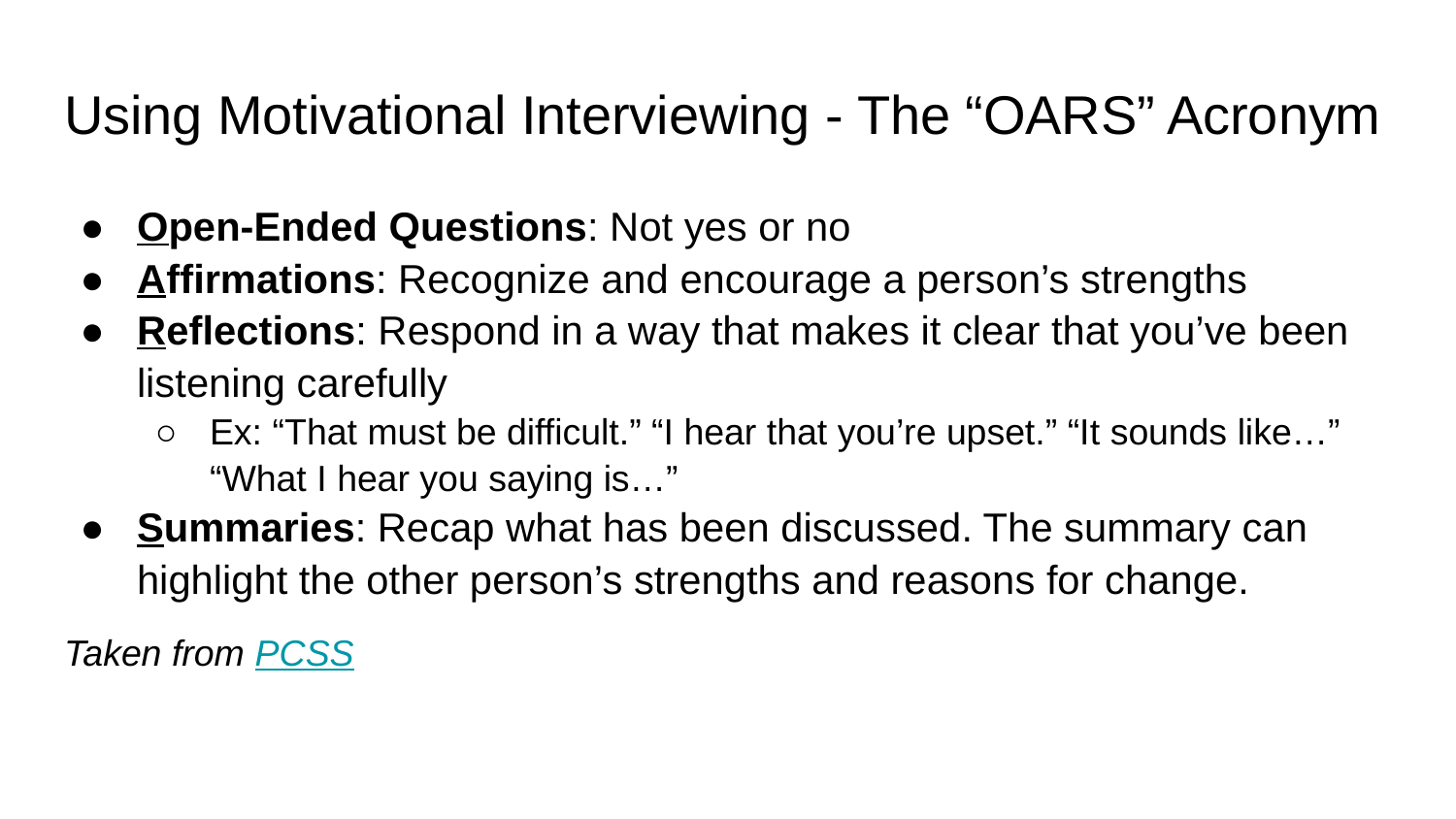

# Using Motivational Interviewing - The “OARS” Acronym
Open-Ended Questions: Not yes or no
Affirmations: Recognize and encourage a person’s strengths
Reflections: Respond in a way that makes it clear that you’ve been listening carefully
Ex: “That must be difficult.” “I hear that you’re upset.” “It sounds like…” “What I hear you saying is…”
Summaries: Recap what has been discussed. The summary can highlight the other person’s strengths and reasons for change.
Taken from PCSS

## Slide 20
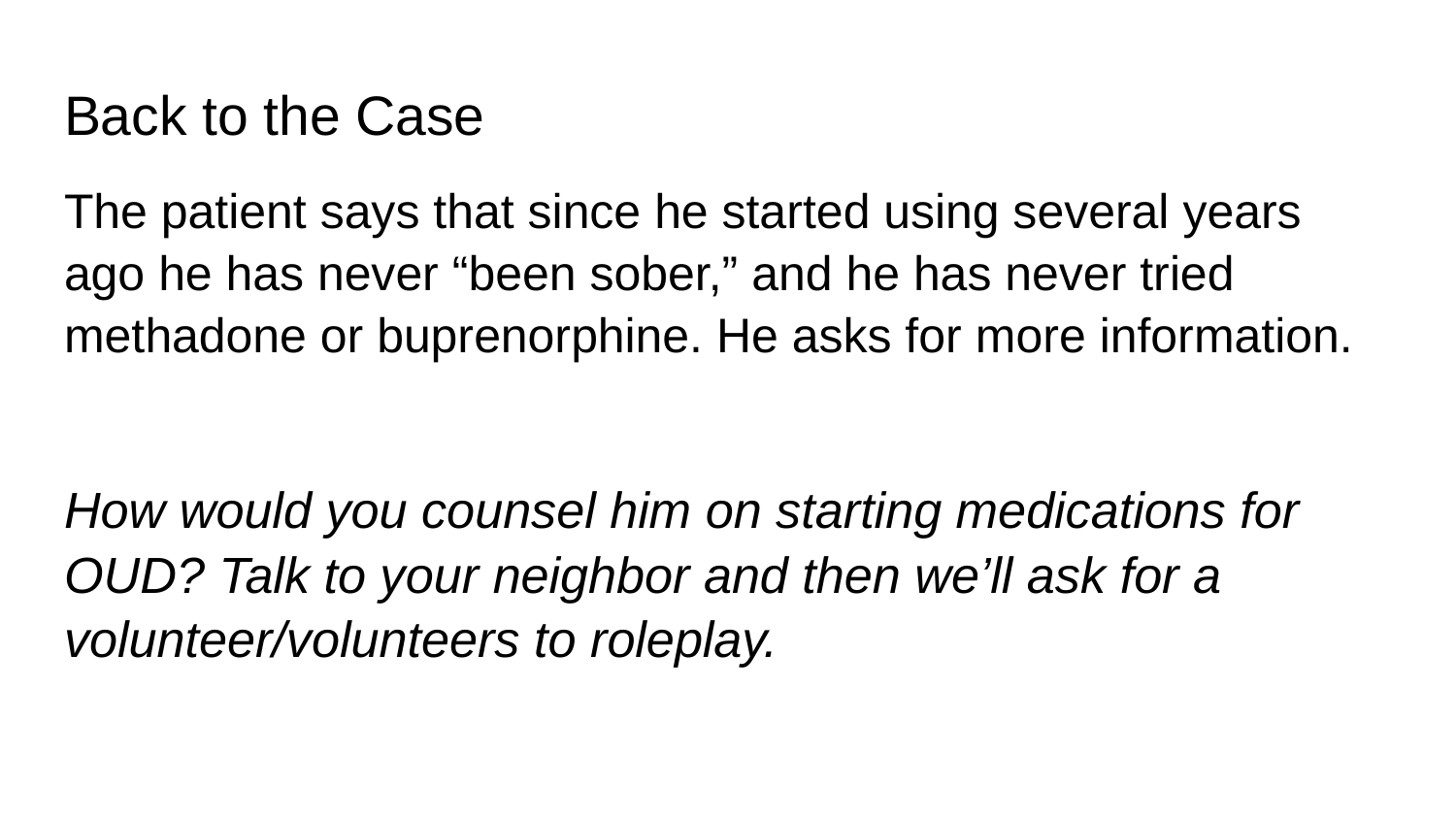

# Back to the Case
The patient says that since he started using several years ago he has never “been sober,” and he has never tried methadone or buprenorphine. He asks for more information.
How would you counsel him on starting medications for OUD? Talk to your neighbor and then we’ll ask for a volunteer/volunteers to roleplay.

## Slide 21
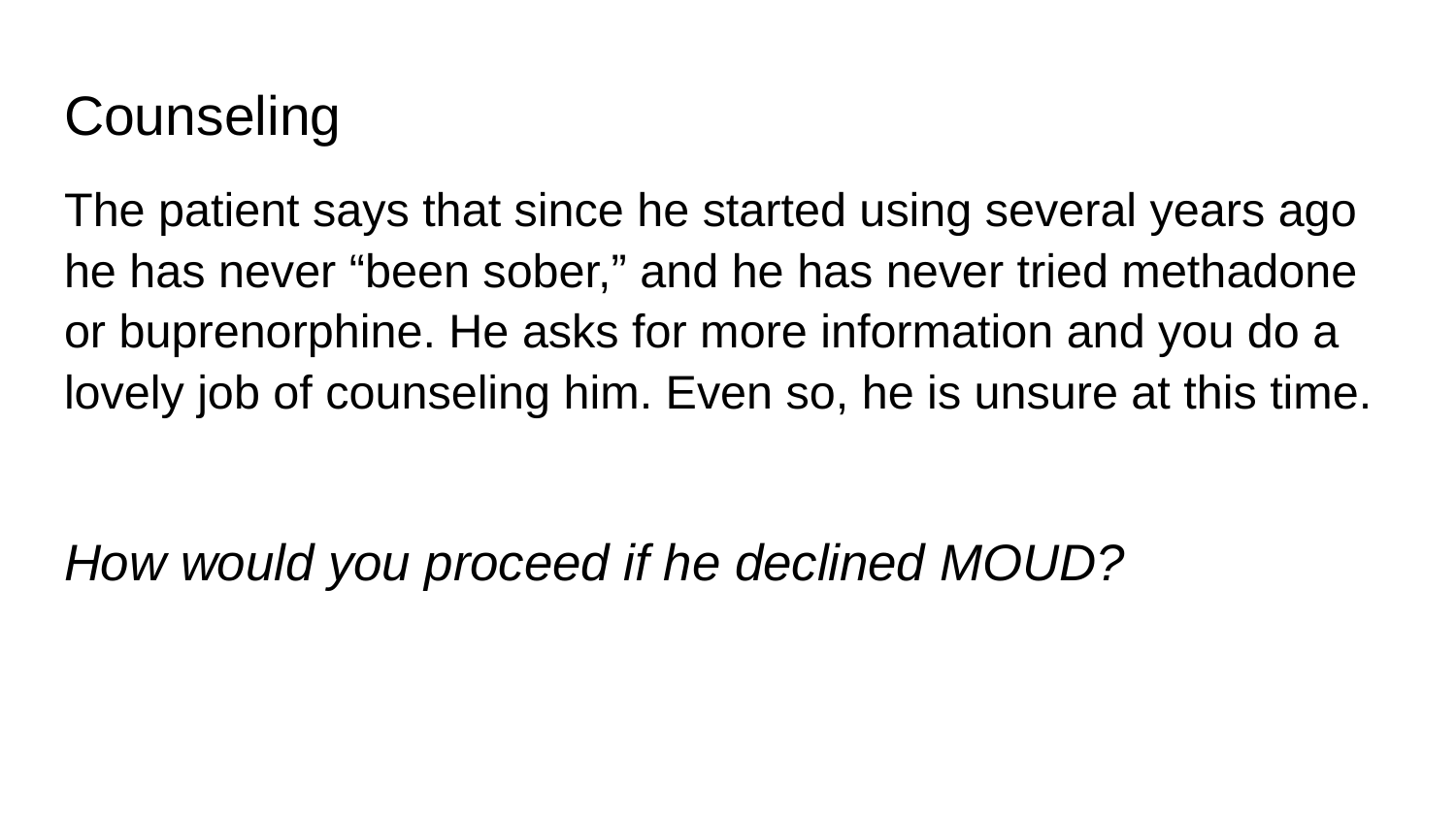

# Counseling
The patient says that since he started using several years ago he has never “been sober,” and he has never tried methadone or buprenorphine. He asks for more information and you do a lovely job of counseling him. Even so, he is unsure at this time.
How would you proceed if he declined MOUD?

## Slide 22
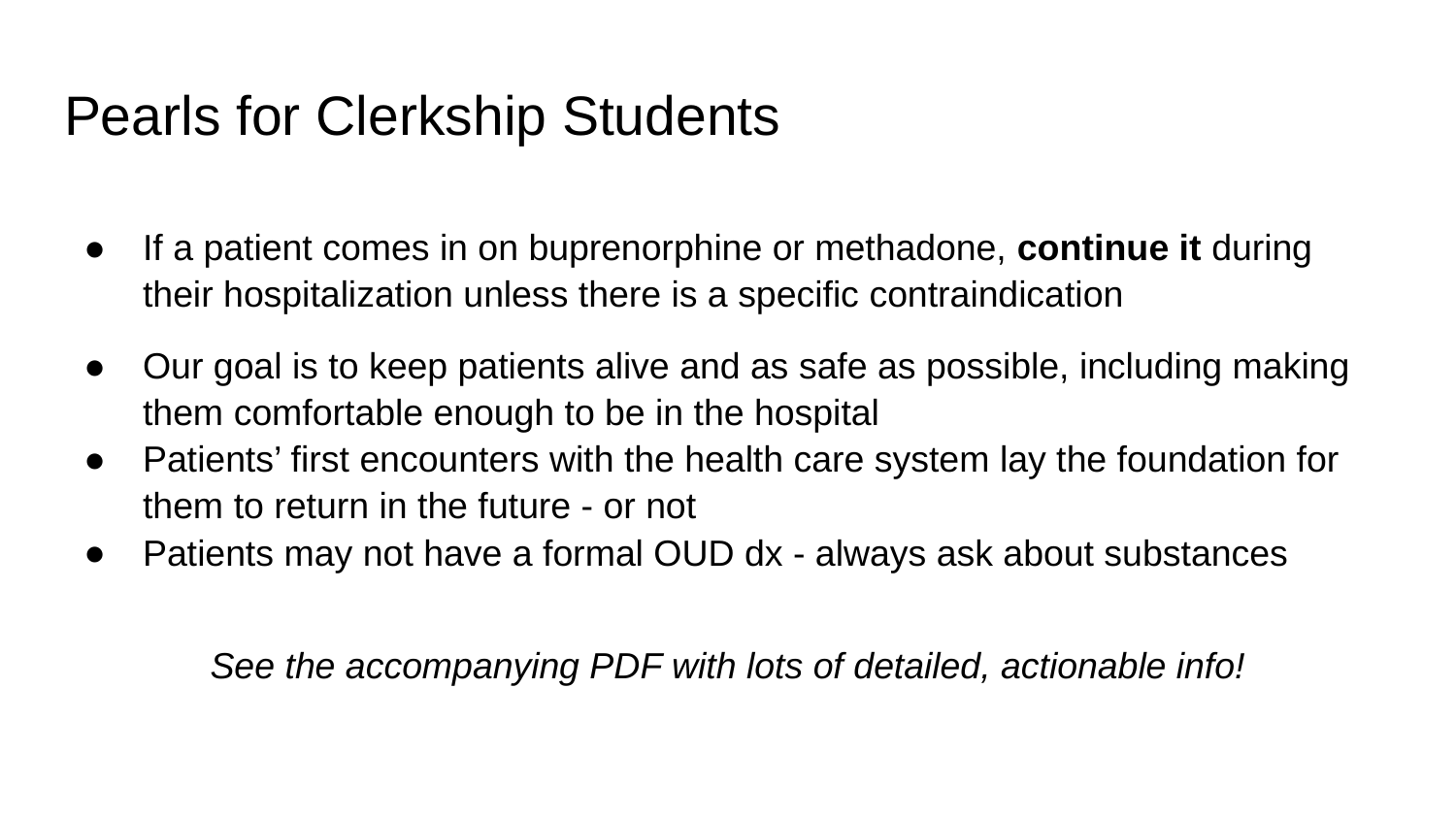

# Pearls for Clerkship Students
If a patient comes in on buprenorphine or methadone, continue it during their hospitalization unless there is a specific contraindication
Our goal is to keep patients alive and as safe as possible, including making them comfortable enough to be in the hospital
Patients’ first encounters with the health care system lay the foundation for them to return in the future - or not
Patients may not have a formal OUD dx - always ask about substances
See the accompanying PDF with lots of detailed, actionable info!

## Slide 23
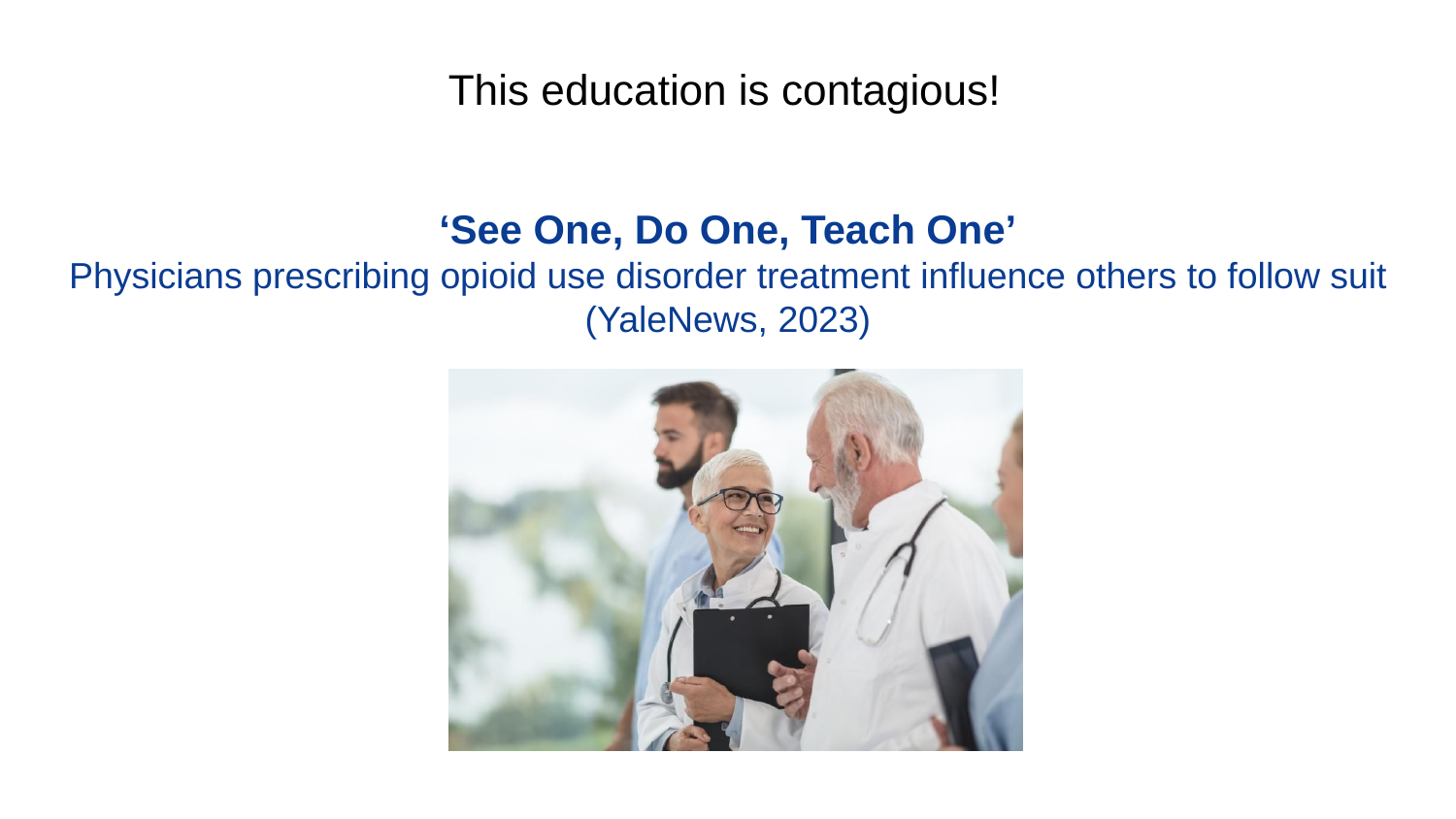

# This education is contagious!
‘See One, Do One, Teach One’
Physicians prescribing opioid use disorder treatment influence others to follow suit (YaleNews, 2023)

## Slide 24
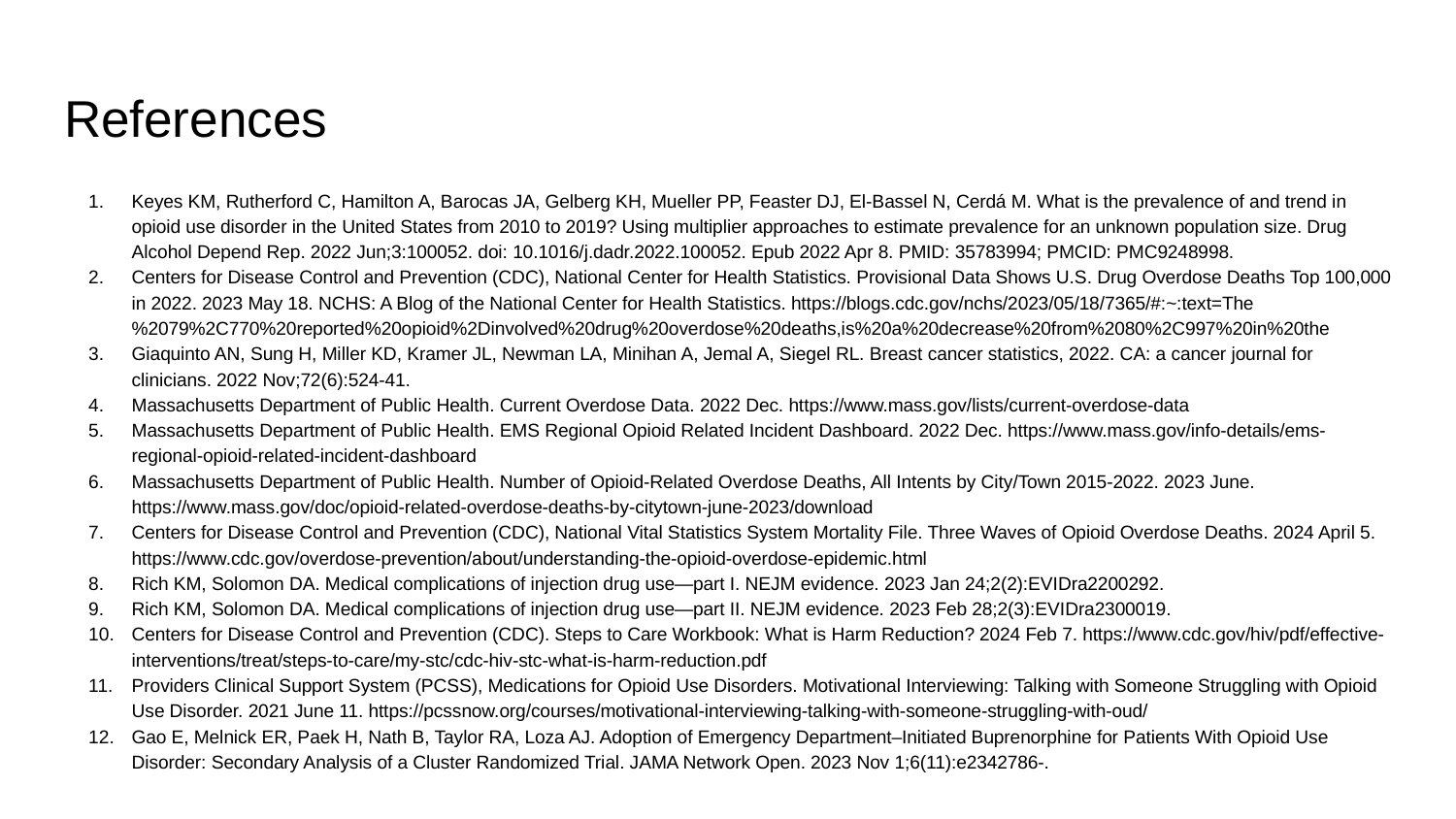

# References
Keyes KM, Rutherford C, Hamilton A, Barocas JA, Gelberg KH, Mueller PP, Feaster DJ, El-Bassel N, Cerdá M. What is the prevalence of and trend in opioid use disorder in the United States from 2010 to 2019? Using multiplier approaches to estimate prevalence for an unknown population size. Drug Alcohol Depend Rep. 2022 Jun;3:100052. doi: 10.1016/j.dadr.2022.100052. Epub 2022 Apr 8. PMID: 35783994; PMCID: PMC9248998.
Centers for Disease Control and Prevention (CDC), National Center for Health Statistics. Provisional Data Shows U.S. Drug Overdose Deaths Top 100,000 in 2022. 2023 May 18. NCHS: A Blog of the National Center for Health Statistics. https://blogs.cdc.gov/nchs/2023/05/18/7365/#:~:text=The%2079%2C770%20reported%20opioid%2Dinvolved%20drug%20overdose%20deaths,is%20a%20decrease%20from%2080%2C997%20in%20the
Giaquinto AN, Sung H, Miller KD, Kramer JL, Newman LA, Minihan A, Jemal A, Siegel RL. Breast cancer statistics, 2022. CA: a cancer journal for clinicians. 2022 Nov;72(6):524-41.
Massachusetts Department of Public Health. Current Overdose Data. 2022 Dec. https://www.mass.gov/lists/current-overdose-data
Massachusetts Department of Public Health. EMS Regional Opioid Related Incident Dashboard. 2022 Dec. https://www.mass.gov/info-details/ems-regional-opioid-related-incident-dashboard
Massachusetts Department of Public Health. Number of Opioid-Related Overdose Deaths, All Intents by City/Town 2015-2022. 2023 June. https://www.mass.gov/doc/opioid-related-overdose-deaths-by-citytown-june-2023/download
Centers for Disease Control and Prevention (CDC), National Vital Statistics System Mortality File. Three Waves of Opioid Overdose Deaths. 2024 April 5. https://www.cdc.gov/overdose-prevention/about/understanding-the-opioid-overdose-epidemic.html
Rich KM, Solomon DA. Medical complications of injection drug use—part I. NEJM evidence. 2023 Jan 24;2(2):EVIDra2200292.
Rich KM, Solomon DA. Medical complications of injection drug use—part II. NEJM evidence. 2023 Feb 28;2(3):EVIDra2300019.
Centers for Disease Control and Prevention (CDC). Steps to Care Workbook: What is Harm Reduction? 2024 Feb 7. https://www.cdc.gov/hiv/pdf/effective-interventions/treat/steps-to-care/my-stc/cdc-hiv-stc-what-is-harm-reduction.pdf
Providers Clinical Support System (PCSS), Medications for Opioid Use Disorders. Motivational Interviewing: Talking with Someone Struggling with Opioid Use Disorder. 2021 June 11. https://pcssnow.org/courses/motivational-interviewing-talking-with-someone-struggling-with-oud/
Gao E, Melnick ER, Paek H, Nath B, Taylor RA, Loza AJ. Adoption of Emergency Department–Initiated Buprenorphine for Patients With Opioid Use Disorder: Secondary Analysis of a Cluster Randomized Trial. JAMA Network Open. 2023 Nov 1;6(11):e2342786-.
