# Supplementary material for: Caring for Patients with Opioid Use Disorder: A Near-Peer Workshop for Medical Students
Source: MedEdPORTAL. 2026 Jan 27;22:11573. doi: 10.15766/mep_2374-8265.11573 (PMC12835442; doi:10.15766/mep_2374-8265.11573)
Supplement: Supplementary file 1 — Presentation Materials.pptxPostsession Handout.docxFacilitator Guide.docxSurveys.docx [file mep_2374-8265.11573-s001.zip › B. Postsession Handout.docx]

***Opioids in the Hospital - Quick Sheet for Medicine Clerkship Students***

**Opioids for Acute Pain**

| ***Commonly Used Opioids on the Medicine Floor*** | | |
| --- | --- | --- |
| **Generic (Brand, if applicable)** | **Route of admin** | **Considerations** |
| *Short-acting/immediate-release formulations* | | |
| **Morphine** | PO, IV, PCA | Renally cleared; consider carefully in AKI or CKD |
| **Oxycodone** | PO | Avoid defaulting to oxycodone-paracetamol (Percocet) formulation |
| **Hydromorphone (Dilaudid)** | PO, IV, SQ, IM, PCA | Potent, fast-acting |
| *Extended-release formulations* | | |
| **Fentanyl*** | Transdermal patch | Patients with chronic pain may use a patch - need to factor in when dosing other meds |

- Used for severe pain along with multimodal agents (NSAIDs, acetaminophen, lidocaine patches)
- Mechanism: mu-opioid receptor agonists
- Contraindications/Considerations: Careful with other sedating medications, e.g. benzodiazepines, first-generation antihistamines; consider prior patient use in exact choice, dosing, precautions, and bridging to care after hospitalization
- What to watch for: respiratory depression, altered mental status, constipation.
- Reverse overdose with naloxone (**Narcan)**
- Always use **immediate-release** formulations for acute pain, rather than extended-release.
- **DynaMed** is a good resource for calculating **equivalent doses** between opioids.
- Always **taper** **gradually.**
- IV opioids can be used in patient-controlled analgesia (**PCA**) - a pump device with a basal rate and bolus that the patient controls by pushing a button, used for very severe pain. The pain team will help!

**Acute Pain Management Pearls for Patients with OUD**

**Fentanyl is also short-acting, often used in surgical or ICU settings in IV form, beyond our scope here*

| **Generic (Brand, if applicable)** | **Description** | **Advantages** | **Disadvantages** |
| --- | --- | --- | --- |
| **Methadone** | Full opioid receptor agonist  - Long-acting  - Must be dispensed from licensed clinics  - Maintenance medication | - Patients may benefit from a high-touch environment  - Patients may prefer full agonists | - Daily clinic is very limiting  - Patients often report stigmatizing or coercive practices |
| **Buprenorphine-naloxone (Suboxone)** | Partial opioid receptor agonist  - High receptor affinity  - Quicker onset compared to methadone  - Maintenance medication | - Not attached to clinics, feel like any other patient  - Has long-acting injectable  - New *microdosing techniques* are withdrawal-sparing! | - Some patients report nausea or dislike the taste  - Risk of precipitated withdrawal with induction |
| **Naltrexone** | Opioid receptor antagonist  - Reduces cravings  - Maintenance medication | - Available for patients who desire no agonists  - Has long-acting injectable | - Patients must undergo withdrawal process prior to initiation - can lead to relapse |
| **Naloxone (Narcan)** | Opioid receptor antagonist  - Quickly reverses overdoses  - Emergency medication | - Crucial to give any patients who use opioids, rx or non-rx | - Causes withdrawal symptoms in immediate aftermath of using |

**Overview of Medications for Opioid Use Disorder**

| **A patient on methadone or buprenorphine should be continued on it for its OUD** **indication** (buprenorphine dose can be lowered in consultation with palliative care and/or acute pain if those services are available) and then use **additional short-acting opioids for pain**. *A patient’s home dose of methadone or buprenorphine is often insufficient for acute pain relief.* |
| --- |
| These patients usually need ***higher* opioid doses for pain**. Patients who use opioids at baseline have **hyperalgesia** (their receptors are more sensitive to pain) as well as having tolerance. |
| A multimodal pain approach (NSAIDs, acetaminophen, hot packs, lidocaine) can ***also*** be used but typically **cannot replace opioids** for pain relief in these patients. For patients in recovery who are concerned about using opioids for pain, **consult the acute pain service** (if available) for additional options (e.g., nerve blocks). |
| The goal is **sufficient pain relief that patients can stay to receive care**. |

**Opioid Withdrawal**

| **Substance use pattern** | **Substance use source** | **History of treatment**  **History Questions for Patients with OUD** | **History of illnesses related to use** | **Social history** |
| --- | --- | --- | --- | --- |
| - How much - How often - How long - Using alone - Sharing needles - Other substances - Cleaning materials - Injection sites - Has naloxone (Narcan) | - Source for substances - Source for needles | - Hx of treatment programs - Hx of recovery period (“sober,” “clean”) - Hx of medications for OUD | - Hospitalizations - Overdoses - Received naloxone (Narcan) - HIV hx - PrEP hx - Hep C hx | - Housing status - Work status - Insurance status - Sexual history |

- Signs and sx of withdrawal: elevated HR, diaphoresis, restlessness, mydriasis, myalgias, rhinorrhea, yawning, tremor, GI upset, anxiety, piloerection (make up the Clinical Opioid Withdrawal Score, “COWS,” that nursing uses)
- Patients will often *tell you they are withdrawing and what has worked for them in the past*. Take this seriously!
- Patients may not be in withdrawal when you meet them, but they are at very high risk for developing it - *make a plan as part of your initial assessment.*
- Managing withdrawal: best practice is to **use buprenorphine or methadone** per American Society of Addiction Medicine
  - If + clinical signs of withdrawal: start sublingual buprenorphine at 2-4 mg or oral methadone at 20 mg
  - If - clinical signs of withdrawal: start microdosing buprenorphine protocol (depends on institution - consult addiction medicine if available and check policy) or oral methadone at 20 mg
  - Adjunct: clonidine 0.1-0.3 mg every 6-8  hours
  - **Reexamine 2-4 hours later.** For sublingual buprenorphine, an increase by 2-4 mg every 4 hours is appropriate, frequently landing at 16-24 mg daily. Oral methadone peaks after 4 hours and then can be increased by 10 mg, typically to 30 mg daily.
  - The next day’s standing dose should incorporate the amount given “as needed” the previous day.

**Opioid Overdose**

- Signs of overdose: somnolence, respiratory depression, miosis.
- Anyone who is unresponsive with +pulse and -breath sounds/respiratory depression should get ***intranasal naloxone (Narcan)!***
- **How to administer intranasal naloxone (Narcan):**


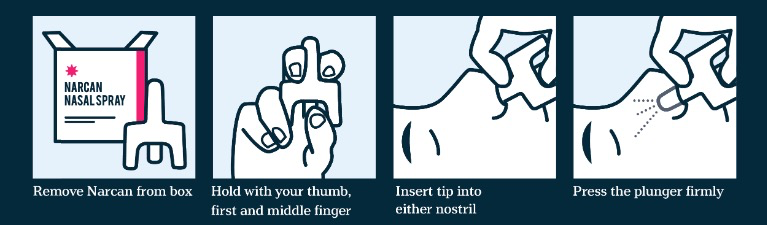


Image by Oshkosh Public Health & WIC, retrieved from https://openstax.org/apps/image-cdn/v1/f=webp/apps/archive/20250916.165151/resources/2bd627278f6a24809995c547d50d441aa9221ebf on 11/27/22025. Image is in the public domain.

**Never Use Alone Hotline: 877-696-1996**

Never Use Alone is a safe, secure, and confidential 24-7 line that provides real-time overdose monitoring via phone and dispatches crisis response when needed.
