# Supplementary material for: Caring for Patients with Opioid Use Disorder: A Near-Peer Workshop for Medical Students
Source: MedEdPORTAL. 2026 Jan 27;22:11573. doi: 10.15766/mep_2374-8265.11573 (PMC12835442; doi:10.15766/mep_2374-8265.11573)
Supplement: Supplementary file 1 — Presentation Materials.pptxPostsession Handout.docxFacilitator Guide.docxSurveys.docx [file mep_2374-8265.11573-s001.zip › C. Facilitator Guide.docx]

**Caring for Patients with Opioid Use Disorder: A Near-Peer Workshop for Medical Students**

*Facilitator Guide*

**Table of Contents**

[Using the Facilitator Guide 0](#_heading=h.k4k2q2cz4hxl)

[Peer Teacher To-Dos Before the Session 0](#_heading=h.2s8eyo1)

[Goals and Objectives 1](#_heading=h.17dp8vu)

[Workshop Outline 2](#_heading=h.3rdcrjn)

[*Section 1 (10-12 minutes): epidemiology 2*](#_heading=h.26in1rg)

[*Section 2 (15 minutes): opioids, OUD diagnosis and management 3*](#_heading=h.lnxbz9)

[*Section 3 (30 minutes): interactive case 5*](#_heading=h.35nkun2)

[*Section 4 (5 min): session summary 8*](#_heading=h.1ksv4uv)

# Using the Facilitator Guide

The following document details how to prepare for and facilitate the “Caring for Patients with Opioid Use Disorder” workshop as a near-peer educator. This Facilitator Guide should be reviewed prior to each workshop so that peer educators can refresh their understanding of the workshop’s objectives and content. The near-peer teachers’ responsibilities prior to each workshop include printing the surveys (these can also be skipped or modified to be administered online depending on the specific institution’s goals for the workshop) and preparing a didactic space in which to present the workshop content (typically all that is needed is a private room with projector/screen-sharing capabilities). Additionally, this Facilitator Guide provides an in-depth outline of the workshop by slide, with suggestions for speaker notes that align with the notes also recorded in Appendix A (Presentation Materials.pptx).

# Peer Teacher To-Dos Before the Session

*Printing the Surveys*

- Can ask Medicine clerkship leadership about the specific number of students in cohort to estimate the number of surveys to print.

*Session*

- Arrive early to place pre-session surveys at students’ seats so they can work on them as they come in.
- Make note of students that arrive late or don’t complete pre-session survey prior to session starting––these surveys should be excluded from analysis if there is a chance that the answers could have been influenced by the workshop.

#

# Goals and Objectives

*Goal:* To provide learners in their Medicine Clerkship with the fundamentals of opioid use disorder (OUD) epidemiology, diagnosis, and management with an emphasis on harm reduction.

*Objectives:*

By the end of this workshop, learners will be able to:

1. Compare and contrast the mechanisms of action of methadone, buprenorphine, naltrexone, and naloxone.
2. Explain three basic principles of managing acute pain for patients who use opioids.
3. Identify three examples of effective harm reduction in a clinical case involving opioid use disorder and injection drug use.
4. Demonstrate a positive shift in attitudes and confidence in managing care for hospitalized patients who use opioids.

*Session design:* 60-minute workshop, in-person.

#

# Workshop Outline

*Note:* **Bolded text represents a new slide.**

## *Section 1 (10-12 minutes): epidemiology*

*(Goal: allow learners to ease into workshop!)*

- **Welcome (5 min):** introductions by near-peer teachers
  - Pre-session survey: handed out as students walk in
  - Preview that first half will be less interactive, second half will be more interactive
- **Group experiences (2 min):**

1. “Raise your hand if you know of anyone, either directly or indirectly, affected by opioid use disorder, including outside of your clinical rotations”
2. “Raise your hand if you have ever had a patient who used opioids of any kind”
3. “Raise your hand if you have ever had a patient whose opioid use was a direct cause of their hospitalization”
4. “Raise your hand if you or your team have ever started a patient on methadone or suboxone”
5. “Raise your hand if you have ever had a patient who was in stable recovery from opioid use disorder”

| **Goal**: create buy-in, establish interactivity right away; develop a framework of acuity vs recovery (i.e., we see a lot of one and not always the other) |
| --- |

- The Opioid Epidemic in 2022
  - **Epidemic scale (2 min)**: Various estimates put the [prevalence](https://pmc.ncbi.nlm.nih.gov/articles/PMC9248998/#:~:text=Approximately%206.7%20to%207.6%20million%20adults%20in,to%20estimate%20the%20prevalence%20of%20OUD%20require) of Americans with OUD between 2% and 5%.
    - [US](https://blogs.cdc.gov/nchs/2023/05/18/7365/#:~:text=The%2079%2C770%20reported%20opioid%2Dinvolved%20drug%20overdose%20deaths,is%20a%20decrease%20from%2080%2C997%20in%20the): Nearly 80,000 people died of an opioid overdose in 2022, mostly from synthetic opioids such as fentanyl (almost [double](https://acsjournals.onlinelibrary.wiley.com/doi/10.3322/caac.21754#:~:text=Estimated%20Cases%20and%20Deaths%20in,will%20die%20from%20breast%20cancer.) the number of breast cancer deaths).
    - [Massachusetts](https://www.mass.gov/lists/current-overdose-data): 2,357 people died of an opioid overdose in 2022, up 2.5% from 2021 (fentanyl in 93%). Nearly 19,000 [EMS calls](https://www.mass.gov/info-details/ems-regional-opioid-related-incident-dashboard) were related to opioids in 2022.
    - [Boston](about:blank): 247 people died of an opioid overdose in 2022. Over 2,600 EMS calls were related to opioids.

| **Goal**: display relevance to students’ practice as they will all take care of these patients |
| --- |

- **Fentanyl deaths (2 min):**
  - Emphasize that the overdose public health crisis has revolved around fentanyl for the past 10 years, not so much heroin or prescription opioids (synthetic opioids represent ‘Wave 3’ of the epidemic).
  - Graphic: [3 waves of overdose deaths](https://www.cdc.gov/overdose-prevention/about/understanding-the-opioid-overdose-epidemic.html) (CDC)

| **Goal**: focus on most relevant epidemiology for this talk without rehashing info students already know |
| --- |

## *Section 2 (15 minutes): opioids, OUD diagnosis and management*

*(Goal: introduce clinical topics with more learner engagement in build-up to case)*

- Giving Opioids in the Hospital
  - **General opioid management in the hospital (4 min):**
    - Mechanism of action: mu-opioid receptor agonists
    - Considerations: Careful with other sedating medications, e.g. benzodiazepines, first-generation antihistamines; need to consider prior patient use in exact choice, dosing, precautions, and bridging to care after hospitalization
    - What to watch for: respiratory depression, altered mental status, constipation. Reverse overdose with intranasal naloxone.
    - CDC guidance on prescribing opioids: “lowest effective dose of short-acting opioids in a quantity no greater than needed for the expected duration of pain severe enough to require opioids”

| **Goal**: remind learners of basic opioid pharmacology and common complications |
| --- |

- Common Medical Concerns for Patients with OUD
  - **Medical concerns (3 min):** info slide on common medical reasons people who inject drugs come to hospitals ([Rich & Solomon 1](https://evidence.nejm.org/doi/full/10.1056/EVIDra2200292), [Rich & Solomon 2](https://evidence.nejm.org/doi/full/10.1056/EVIDra2300019))
    - Infections - often very painful
      - Bacterial: skin and soft tissue infection, endocarditis, septic arthritis, osteomyelitis, pneumonia
      - Viral: HIV, HCV, HBV, HAV if severe
    - Wounds (e.g. from xylazine) - often very painful
    - *Common OUD comorbidities*: depression, anxiety, chronic venous insufficiency, renal injury, pulmonary hypertension, vascular disease, other SUDs - i.e. alcohol, cocaine
- Helping Patients
  - **Harm reduction principles (2 min):**
    - Definition: “Any behavior or strategy that helps reduce risk or harm to the patient or others.” ([CDC](about:blank))
    - Caring for patients with diabetes as analogy - physicians don’t recommend completely cutting out soda and carbs and then punishing patients if they aren’t able to adhere to this recommendation. They negotiate with patients to find realistic goals, replace soda with water where able, and start medications like metformin when necessary.
    - For substance use disorders, examples of harm reduction include: giving naloxone (Narcan) to reverse opioid overdose, safer use supplies to prevent complications of injecting drugs, and prescribing medications for OUD.

| **Goal**: review the definition and relevance of harm reduction |
| --- |

- **Medications for OUD (2 min):** informational slide, tell learners they will get more info in a post-session handout. Clarify that our goal for this content is to broadly introduce medications for OUD and overdose reversal and to normalize them as evidence-based treatments in supporting patients (goal is NOT to cover selection and dosing strategies).

*For maintenance OUD treatment...*

- - Methadone: slow and long-acting full agonist, attached to special clinics (unless prescription indication is pain)
    - Advantages: some patients benefit from a high-touch environment; some patients prefer full agonists
    - Disadvantages: clinic is very limiting (have to go daily, maybe can get days to weeks depending), patients often report stigmatizing or coercive practices
  - Buprenorphine: partial agonist with high receptor affinity, quicker-acting
    - Advantages: not attached to clinics, allows patients to feel like any other patient; comes in long-acting injectable
    - Disadvantages: some patients report nausea or dislike the taste, risk of precipitated withdrawal with standard induction but, *importantly*, we now have microdosing techniques that are withdrawal-sparing
  - Naltrexone: antagonist that can reduce cravings, used as maintenance medication; must go through withdrawal before initiating

*For overdose reversal...*

- - Naloxone (Narcan): antagonist that quickly reverses overdoses, a crucial part of harm reduction to give to any patient who uses opioids (prescription or non-prescription)

| **Goal**: compare and contrast the mechanisms and common indications for methadone, buprenorphine, naltrexone, and naloxone (Narcan) |
| --- |

- **Acute pain management in OUD (4 min):**
  - 1. The goal is sufficient pain relief that they can stay to receive care without being sleepy or having respiratory depression.
  - 2. A patient on methadone or buprenorphine should be continued on it for MOUD (buprenorphine dose can be lowered in consultation with pall care/acute pain) and you can consider additional short-acting opioids (like morphine, hydromorphone, oxycodone) as well for their pain - while a patient’s home dose of methadone or buprenorphine can help with pain, it is often insufficient for acute pain relief.
  - 3. These patients usually need higher opioid doses for pain. Patients who use opioids at baseline have “hyperalgesia” - their receptors are more sensitive to pain - as well as having a tolerance that other patients do not have.
  - 4. A multimodal pain approach can also be used (acetaminophen, ibuprofen, hot packs) but typically cannot replace opioids for pain relief with these patients.

| **Goal**: provide informational slide on clinical pearls for acute pain management in OUD (also part of the post-session handout for students) |
| --- |

## *Section 3 (30 minutes): interactive case*

*(Goal: encode clinical pearls through engagement)*

*Learner volunteer to read*...

- **Case (7-8 min): “**A 25-year-old man is admitted with a painful, red wound on his left arm. He reports that he injected fentanyl this morning.”
  - “What are 3 examples of history questions to ask this patient, in addition to questions about the presenting concern of the wound?”
  - *Please note that cases like this often veer into stereotypes, and so this case adheres closely to a real patient of a medical student who made the curriculum, who shared similarities to other patients of hers who used drugs.*
  - *Note on language:* remember to be thoughtful, objective, and harm-reduction-oriented when writing notes! A patient “has OUD,” “uses fentanyl,” “uses multiple substances,” “has been in recovery” rather than “is an addict,” “abuses drugs,” “is sober.”

| **Substance use pattern** | **Substance use source** | **History of treatment** | **History of illnesses related to use** | **Social history** |
| --- | --- | --- | --- | --- |
| - How much - How often - How long - Using alone - Sharing needles - Using other substances - Cleaning materials - Injection sites - Has naloxone (Narcan) | - Source for substances - Source for needles | - Hx of treatment programs - Hx of recovery period (“sober,” “clean”) - Hx of medications for OUD | - Hospitalizations - Overdoses - Received naloxone (Narcan) - HIV hx - PrEP hx - Hep C hx | - Housing status - Work status - Insurance status - Sexual history |

| **Goal**: increase interactivity, use clinical case to demonstrate harm reduction and MOUD principles, build history, documentation, and counseling skills |
| --- |

*Learner volunteer to read*...

- **History (4 min): “**The patient reports that he uses around 3 grams of fentanyl daily, always with new needles and works that he cleans with bleach. He typically uses with his partner and has never overdosed, although his partner recently did, which required him to reverse her overdose with naloxone (Narcan). He has more naloxone (Narcan**)** at home. His wound is the site he had been using to inject recently and he has been hospitalized twice for similar skin infections.”
  - “What are 3 ways that this patient is engaging in harm reduction?”
    - *Possible answers:* uses new needles, cleans materials with bleach (rather than tap water), doesn’t use alone, carries and knows how to use naloxone (Narcan), has sought medical care in the past

| **Goal**: reframe the patient’s behaviors from “risky” to focus on his attempts to keep himself and his community safe within his parameters |
| --- |

*Peer teacher to read...*

- **Physical exam (2 min):** “What aspects of the physical exam would you prioritize for this patient?”
  - *Important systems on exam:* vitals (systemic infection), HEENOT for dental and mucositis, cardiac exam for murmurs (endocarditis), pulmonary exam (pneumonia, septic emboli), full skin exam (wounds, phlebitis)
  - For this patient, can point out diaphoresis, fever, dry mucous membranes, normal pupil size, normal cardiopulmonary exam, and evidence of skin and soft tissue infection as key findings.

*Learner volunteer to read...*

- **Assessment (3 min): “**25-year-old man with daily injected fentanyl use presenting with a painful, erythematous area surrounding an open wound on his left arm, found to have fever to 102F.”
  - “What are the top 3 items on your problem list?”
    - *Possible answers:* Cellulitis (likely related to substance use), possible systemic infection, acute pain from infection, active fentanyl use disorder
- **Primer on motivational interviewing (3 min):**
  - *Ask learners what their background with MI is! They may have been introduced to it before, which will save time in the explanation.*
  - MI is a kindness-based, strengths-based, and evidence-based approach to help patients find, strengthen, and act on their own motivations to change. An effective, non-judgmental approach to communication is important for *all* patients!
  - OARS acronym, [PCSS](https://pcssnow.org/courses/motivational-interviewing-talking-with-someone-struggling-with-oud/)
    - *Open-Ended Questions*: Ask questions that can’t be answered with yes or no.
    - *Affirmations*: Recognize and encourage a person’s strengths!
    - *Reflections*: Respond in a way that makes it obvious that you’ve been listening carefully. The other person can then make corrections if they did not express themselves fully. This also allows the listener to express “empathy,” the ability to see the world through another’s eyes and share in their feelings and experiences. This can make the other person feel heard and understood.
      - *Ex: “That must be difficult.” “I hear that you’re upset.” “It sounds like…” “What I hear you saying is…”*
    - *Summaries*: Summaries allow the listener to “recap” what has been discussed. The summary can highlight the other person’s strengths and reasons for change.

| **Goal:** define motivational interviewing and introduce a strategy to apply to interviewing |
| --- |

*Learner volunteer to read...*

- **Counseling (10 min):** “The patient says that since he started using several years ago he has never ‘been sober,’ and he has never tried methadone or buprenorphine. He asks for more information.”
  - “How would you counsel him on starting medications for OUD? Talk to your neighbor and then we’ll ask for a volunteer to role-play.” *Think-pair-share followed by a volunteer/several volunteers to roleplay (5 min to discuss, 5 min roleplay with near-peer teacher as patient).*
  - *Note:* Acknowledge that roleplays can be stressful and state that it’s okay if a few students just try asking 1-2 questions. Emphasize that it’s helpful to say these questions out loud in a safe environment before having these conversations with a patient.

| Roleplay: One or more students will volunteer to interview a peer teacher, who will act as the patient. Some examples of questions students may ask are listed below (these questions can also be mentioned after the roleplay as examples of ways to use motivational interviewing techniques).  There is not a ‘script’ to follow as the patient, however, peer teachers acting as the patient can utilize the following information in their answers:   - Patient has been using fentanyl for several years. - The experience of having their partner overdose and being the one to reverse them was frightening. The patient has always known that using opioids in this way is risky, but the experience was a wake-up call. - Patient has been interested in seeking treatment in the past, but has heard bad experiences from friends who were traveling to methadone clinics daily to receive treatment. |
| --- |

Example language with key points:

- *Questions*:

> Is it okay if we take a minute to talk about your fentanyl use (*asking permission*)?

> What makes you interested in learning more about treatment options (*open-ended question to elicit motivations for change*)?

> What are some aspects of using fentanyl that you like? What are some aspects of using that you’re not happy with (*exploring pros and cons in patient’s own words*)?

> What may happen if things continue as they are? What would be different if you went for treatment (*looking forward*)?

- *Key counseling points related to treatment options*:

> Methadone would be dispensed at a clinic that you would go to every morning; Suboxone could be prescribed like other medications.

> We would start either medication here in the hospital so we can watch how you’re doing and get you to a dose where you feel comfortable enough to be in recovery from fentanyl.

> We can start slow with either medication so that you don’t feel any withdrawal. Our goal is always to keep you comfortable and safe, and avoid you feeling withdrawal symptoms.

> We will make sure that we have a plan for continuing the medication after you leave the hospital and give you more naloxone (Narcan).

*Learner volunteer to read***...**

- **Counseling cont. (2 min): “**The patient says that since he started using several years ago he has never “been sober,” and he has never tried methadone or buprenorphine. He asks for more information and you do a lovely job of counseling him. Even so, he is unsure at this time.”
  - “How would you proceed if he declined MOUD?”

Example language with key points:

- We’re here for you wherever you are right now.

- It sounds like you already have some strategies to keep yourself safe, but would you mind if the team discussed some other strategies with you as well?

- We will also give you more naloxone (Narcan). If you ever change your mind, we are here to support you.

| **Goal:** demonstrate medical counseling and education about medications for opioid use disorder and harm reduction in a roleplay scenario |
| --- |

## *Section 4 (5 min): session summary*

*(Goal: summarize key points of session and importance of this education)*

- **Pearls for clerkship students (3 min):** reiterate that accompanying pdf will have lots of actionable info!
  - If a patient comes in on buprenorphine or methadone, continue it during their hospitalization unless there is a specific contraindication (e.g. QT prolongation).
  - Our goal is to keep patients alive and as safe as possible, including making them comfortable enough to be in the hospital.
  - Patients’ first encounters with the health care system lay the foundation for them to return in the future - or not.
  - Patients may not have a formal OUD diagnosis - always ask about substances.
- **Wrap-up slide (2 min)**:
  - [JAMA Network Open](https://jamanetwork.com/journals/jamanetworkopen/fullarticle/2811640) paper headline from YaleNews highlighting that prescribing treatment for OUD among ED physicians is contagious!

**Peer Teacher To-Dos After the Session**

- Answer any questions
- Post-session survey: handed out before students leave
- End-of-clerkship survey: distributed in-person or electronically during the final week of the clerkship.
