# Supplementary material for: Caring for Patients with Opioid Use Disorder: A Near-Peer Workshop for Medical Students
Source: MedEdPORTAL. 2026 Jan 27;22:11573. doi: 10.15766/mep_2374-8265.11573 (PMC12835442; doi:10.15766/mep_2374-8265.11573)
Supplement: Supplementary file 1 — Presentation Materials.pptxPostsession Handout.docxFacilitator Guide.docxSurveys.docx [file mep_2374-8265.11573-s001.zip › D. Surveys.docx]

| **PRESURVEY** | **Last 4 digits of phone number (to link all surveys): __________** |
| --- | --- |

Responses to this survey are anonymous. Your answers to the following questions will not be tracked by the Medicine Clerkship leadership and will not impact your Clerkship evaluation. Survey responses will, however, be analyzed in aggregate to evaluate the session for the purposes of improving it for future classes and for publishing an article for public dissemination. We ask for the last 4 digits of your phone number only to link all responses. This should take no longer than 10 minutes. Thank you!

1.

|  | Strongly disagree | Disagree | Not sure but probably disagree | Not sure but probably agree | Agree | Strongly agree |
| --- | --- | --- | --- | --- | --- | --- |
| I prefer not to work with patients with opioid use disorder. |  |  |  |  |  |  |
| Patients with opioid use disorder irritate me. |  |  |  |  |  |  |
| I enjoy giving extra time to patients with opioid use disorder. |  |  |  |  |  |  |
| Patients with opioid use disorder are particularly difficult for me to work with. |  |  |  |  |  |  |
| Working with patients with opioid use disorder is satisfying. |  |  |  |  |  |  |
| I feel especially compassionate toward patients with opioid use disorder. |  |  |  |  |  |  |
| I wouldn’t mind getting up on call nights to care for patients with opioid use disorder. |  |  |  |  |  |  |
| I can usually find something that helps patients with opioid use disorder feel better. |  |  |  |  |  |  |
| There is little I can do to help patients with opioid use disorder. |  |  |  |  |  |  |
| Insurance plans should cover patients with opioid use disorder to the same degree that they cover patients with other conditions. |  |  |  |  |  |  |
| Treating patients with opioid use disorder is an ineffective use of medical dollars. |  |  |  |  |  |  |

2. A 42-year-old woman has severe flank pain secondary to a large ureteric calculus (kidney stone). She is on methadone maintenance treatment for opioid use disorder. Which of the following is the best treatment option for her acute pain?

1. Stop methadone, start max-dose ibuprofen, and give heat packs
2. Decrease methadone dose, give IV morphine as needed
3. Maintain methadone dose, give IV morphine as needed
4. Increase methadone dose
5. Don’t know

3.

|  | True | False | I don’t know |
| --- | --- | --- | --- |
| Long-acting opioids are used to treat chronic “round the clock” pain. |  |  |  |
| Methadone is a long-acting opioid. |  |  |  |
| Restlessness, muscle and bone pain, and insomnia are symptoms of opioid withdrawal. |  |  |  |
| Heroin, oxycontin, and fentanyl are all examples of opioids. |  |  |  |
| Trouble breathing is NOT related to opioid overdose. |  |  |  |
| Clammy and cool skin is NOT a sign of an opioid overdose. |  |  |  |
| All overdoses are fatal (deadly). |  |  |  |
| Using a short-acting opioid and a long-acting opioid at the same time does NOT increase your risk of an opioid overdose. |  |  |  |
| If you see a person overdosing on opioids, you can begin rescue breathing until a health worker arrives. |  |  |  |
| A sternal rub helps you evaluate whether someone is unconscious. |  |  |  |
| Once you confirm an individual is breathing, you can place him/her into the recovery position. |  |  |  |
| Narcan (naloxone) will reverse the effect of an opioid overdose. |  |  |  |

4.

|  | Strongly disagree | Disagree | Neutral | Agree | Strongly agree |
| --- | --- | --- | --- | --- | --- |
| I feel knowledgeable about how best to work with patients with opioid use disorder. |  |  |  |  |  |
|  | | | | | |
| ***How confident are you with...*** | Not at all confident | Slightly confident | Somewhat confident | Very confident | Extremely confident |
| ...describing common medical concerns of patients who inject drugs? |  |  |  |  |  |
| ...describing strategies for acute pain management for patients with OUD? |  |  |  |  |  |
| ...identifying examples of harm reduction in care of patients with OUD? |  |  |  |  |  |
| ...asking patients with OUD about their opioid use? |  |  |  |  |  |
| ...counseling a patient with OUD about initiating medications for OUD in the hospital? |  |  |  |  |  |

| **POSTSURVEY** | **Last 4 digits of phone number (to link pre- and post-surveys): __________** |
| --- | --- |

1.

|  | Strongly disagree | Disagree | Not sure but probably disagree | Not sure but probably agree | Agree | Strongly agree |
| --- | --- | --- | --- | --- | --- | --- |
| I prefer not to work with patients with opioid use disorder. |  |  |  |  |  |  |
| Patients with opioid use disorder irritate me. |  |  |  |  |  |  |
| I enjoy giving extra time to patients with opioid use disorder. |  |  |  |  |  |  |
| Patients with opioid use disorder are particularly difficult for me to work with. |  |  |  |  |  |  |
| Working with patients with opioid use disorder is satisfying. |  |  |  |  |  |  |
| I feel especially compassionate toward patients with opioid use disorder. |  |  |  |  |  |  |
| I wouldn’t mind getting up on call nights to care for patients with opioid use disorder. |  |  |  |  |  |  |
| I can usually find something that helps patients with opioid use disorder feel better. |  |  |  |  |  |  |
| There is little I can do to help patients with opioid use disorder. |  |  |  |  |  |  |
| Insurance plans should cover patients with opioid use disorder to the same degree that they cover patients with other conditions. |  |  |  |  |  |  |
| Treating patients with opioid use disorder is an ineffective use of medical dollars. |  |  |  |  |  |  |

2. A 42-year-old woman has severe flank pain secondary to a large ureteric calculus (kidney stone). She is on methadone maintenance treatment for opioid use disorder. Which of the following is the best treatment option for her acute pain?

1. Stop methadone, start max-dose ibuprofen, and give heat packs
2. Decrease methadone dose, give IV morphine as needed
3. Maintain methadone dose, give IV morphine as needed
4. Increase methadone dose
5. Don’t know

3.

|  | True | False | I don’t know |
| --- | --- | --- | --- |
| Long-acting opioids are used to treat chronic “round the clock” pain. |  |  |  |
| Methadone is a long-acting opioid. |  |  |  |
| Restlessness, muscle and bone pain, and insomnia are symptoms of opioid withdrawal. |  |  |  |
| Heroin, oxycontin, and fentanyl are all examples of opioids. |  |  |  |
| Trouble breathing is NOT related to opioid overdose. |  |  |  |
| Clammy and cool skin is NOT a sign of an opioid overdose. |  |  |  |
| All overdoses are fatal (deadly). |  |  |  |
| Using a short-acting opioid and a long-acting opioid at the same time does NOT increase your risk of an opioid overdose. |  |  |  |
| If you see a person overdosing on opioids, you can begin rescue breathing until a health worker arrives. |  |  |  |
| A sternal rub helps you evaluate whether someone is unconscious. |  |  |  |
| Once you confirm an individual is breathing, you can place him/her into the recovery position. |  |  |  |
| Narcan (naloxone) will reverse the effect of an opioid overdose. |  |  |  |

4.

|  | Strongly disagree | Disagree | Neutral | Agree | Strongly agree |
| --- | --- | --- | --- | --- | --- |
| I feel knowledgeable about how best to work with patients with opioid use disorder. |  |  |  |  |  |
|  | | | | | |
| ***How confident are you with...*** | Not at all confident | Slightly confident | Somewhat confident | Very confident | Extremely confident |
| ...describing common medical concerns of patients who inject drugs? |  |  |  |  |  |
| ...describing strategies for acute pain management for patients with OUD? |  |  |  |  |  |
| ...identifying examples of harm reduction in care of patients with OUD? |  |  |  |  |  |
| ...asking patients with OUD about their opioid use? |  |  |  |  |  |
| ...counseling a patient with OUD about initiating medications for OUD in the hospital? |  |  |  |  |  |

| **END-OF-CLERKSHIP SURVEY** | **Last 4 digits of phone number (to link all surveys): __________** |
| --- | --- |

Responses to this survey are anonymous. Your answers to the following questions will not be tracked by the Medicine Clerkship leadership and will not impact your Clerkship evaluation. Survey responses will, however, be analyzed in aggregate to evaluate the session for the purposes of improving it for future classes and for publishing an article for public dissemination. We ask for the last 4 digits of your phone number only to link all responses. This should take no longer than 10 minutes. Thank you!

1.

|  | Strongly disagree | Disagree | Not sure but probably disagree | Not sure but probably agree | Agree | Strongly agree |
| --- | --- | --- | --- | --- | --- | --- |
| I prefer not to work with patients with opioid use disorder. |  |  |  |  |  |  |
| Patients with opioid use disorder irritate me. |  |  |  |  |  |  |
| I enjoy giving extra time to patients with opioid use disorder. |  |  |  |  |  |  |
| Patients with opioid use disorder are particularly difficult for me to work with. |  |  |  |  |  |  |
| Working with patients with opioid use disorder is satisfying. |  |  |  |  |  |  |
| I feel especially compassionate toward patients with opioid use disorder. |  |  |  |  |  |  |
| I wouldn’t mind getting up on call nights to care for patients with opioid use disorder. |  |  |  |  |  |  |
| I can usually find something that helps patients with opioid use disorder feel better. |  |  |  |  |  |  |
| There is little I can do to help patients with opioid use disorder. |  |  |  |  |  |  |
| Insurance plans should cover patients with opioid use disorder to the same degree that they cover patients with other conditions. |  |  |  |  |  |  |
| Treating patients with opioid use disorder is an ineffective use of medical dollars. |  |  |  |  |  |  |

2.

|  | True | False | I don’t know |
| --- | --- | --- | --- |
| Long-acting opioids are used to treat chronic “round the clock” pain. |  |  |  |
| Methadone is a long-acting opioid. |  |  |  |
| Restlessness, muscle and bone pain, and insomnia are symptoms of opioid withdrawal. |  |  |  |
| Heroin, oxycontin, and fentanyl are all examples of opioids. |  |  |  |
| Trouble breathing is NOT related to opioid overdose. |  |  |  |
| Clammy and cool skin is NOT a sign of an opioid overdose. |  |  |  |
| All overdoses are fatal (deadly). |  |  |  |
| Using a short-acting opioid and a long-acting opioid at the same time does NOT increase your risk of an opioid overdose. |  |  |  |
| If you see a person overdosing on opioids, you can begin rescue breathing until a health worker arrives. |  |  |  |
| A sternal rub helps you evaluate whether someone is unconscious. |  |  |  |
| Once you confirm an individual is breathing, you can place him/her into the recovery position. |  |  |  |
| Narcan (naloxone) will reverse the effect of an opioid overdose. |  |  |  |

3.

|  | Strongly disagree | Disagree | Neutral | Agree | Strongly agree |
| --- | --- | --- | --- | --- | --- |
| I feel knowledgeable about how best to work with patients with opioid use disorder. |  |  |  |  |  |
|  | | | | | |
| ***How confident are you with...*** | Not at all confident | Slightly confident | Somewhat confident | Very confident | Extremely confident |
| ...describing common medical concerns of patients who inject drugs? |  |  |  |  |  |
| ...describing strategies for acute pain management for patients with OUD? |  |  |  |  |  |
| ...identifying examples of harm reduction in care of patients with OUD? |  |  |  |  |  |
| ...asking patients with OUD about their opioid use? |  |  |  |  |  |
| ...counseling a patient with OUD about initiating medications for OUD in the hospital? |  |  |  |  |  |

4. Approximately how many patients with OUD (active or in recovery) were on your inpatient teams throughout the clerkship?
