# Supplementary material for: Caring for Patients with Opioid Use Disorder: A Near-Peer Workshop for Medical Students
Source: MedEdPORTAL. 2026 Jan 27;22:11573. doi: 10.15766/mep_2374-8265.11573 (PMC12835442; doi:10.15766/mep_2374-8265.11573)
Supplement: Supplementary file 1 — Presentation Materials.pptxPostsession Handout.docxFacilitator Guide.docxSurveys.docx [file mep_2374-8265.11573-s001.zip › _Educational Summary Report_11573.pdf]

MedEdPORTAL
THE AAMC JOURNAL OF TEACHING AND LEARNING RESOURCES

Original Publication Open Access

Caring for Patients with Opioid Use Disorder: A Near-Peer Workshop for Medical Students

William C. Oles, MD*, Erika Lynn-Green, MD, MSc, Rory Vu Mather, MS, Michael Motoc, MA, Allison Pellegrino, Hilary S. Connery, MD, PhD, Patrick McGuire, MD, Julia Lindenberg, MD, Kevin P. Hill, MD, MHS, Stephen R. Pelletier, PhD, Ryan E. Nelson, MD, Amy R. Weinstein, MD, MPH

*Corresponding author: william.oles@ucsf.edu

Abstract

Introduction: The opioid epidemic remains a public health crisis driven largely by synthetic opioids like fentanyl. Gaps in undergraduate medical education contribute to health disparities among patients with opioid use disorder (OUD). We developed a peer-led workshop for medical students to enhance their competence and confidence in managing hospitalized patients with OUD. **Methods:** Students participated in a 60-minute, peer-led, case-based workshop during their Medicine Clerkship. Workshop content focused on fentanyl's role in the opioid epidemic, counseling about medication for OUD (MOUD), and acute pain management for patients with OUD. We assessed students' knowledge, attitudes, and confidence in managing patients with OUD with surveys and a focus group. **Results:** Sixty students participated in the workshop. Postworkshop surveys showed a significant increase in confidence asking about opioid use ($p = .02$), counseling about MOUD ($p < .001$), identifying harm reduction practices ($p < .001$), and managing acute pain ($p < .001$). These findings were associated with an improvement in attitudes about patients with OUD and an increase in general knowledge about opioids ($p = .004$). Student feedback affirmed the clinical applicability of the material and supported the peer-led teaching format. **Discussion:** This peer-led workshop enhanced medical students' confidence and competence in caring for hospitalized patients with OUD. Future directions include expanding participation and refining the assessment tools to better capture skill acquisition. Our findings underscore the importance of integrating OUD management education into undergraduate medical education curricula and the key role that near-peer teachers can play in this implementation.

Keywords
Opioid Use Disorder (OUD), Near-Peer Teaching, Medications for Opioid Use Disorder (MOUD), Harm Reduction, Substance Use Disorders, Substance Abuse/Addiction

Educational Objectives

By the end of this workshop, learners will be able to:

1. Identify the medications used to treat opioid use disorder and reverse opioid overdose.
2. Select an acute pain management plan for a patient receiving long-term opioid therapy.
3. Demonstrate a positive shift in attitudes and confidence in managing care for hospitalized patients who use opioids.

Citation:
Oles WC, Lynn-Green E, Mather RV, et al. Caring for patients with opioid use disorder: a near-peer workshop for medical students. *MedEdPORTAL.* 2026;22:11573.
https://doi.org/10.15766/mep_2374-8265.11573

Introduction

The opioid epidemic remains a public health crisis with high rates of morbidity and mortality for those affected. As of September 2023, 12-month drug overdose mortality in the US remained above 100,000 persons, with 70% of deaths attributable to synthetic opioids.[1] Patients who receive medications for opioid use disorder (MOUD) have a mortality rate less than one-third of the rate of their counterparts who are not prescribed MOUD.[2] Despite increased awareness of treatment need, fewer than 20% of patients with opioid use disorder (OUD) are prescribed MOUD each year.[3] System-level strategies to address the epidemic—-such as overdose reversal training, harm reduction programs, and the elimination of prescriber buprenorphine waivers—-aim to reduce opioid-related hospitalization and death. However, given the ongoing underutilization of medications, shortage of addiction specialists, and rising incidence of OUD, there is an urgent need

Copyright © 2026 Oles et al. This is an open-access publication distributed under the terms of the Creative Commons Attribution license.

to empower more clinicians and medical students to evaluate and manage today's OUD crisis.[4,5]

Since 2016, students and educators at Harvard Medical School (HMS) have collaborated within the HMS Substance Use and Pain (SUP) Curriculum Committee to expand teaching on substance use and pain management across all phases of undergraduate medical training. The SUP Committee was initially formed in response to advocacy following a rise in fentanyl overdoses in Massachusetts and has since functioned to maintain the substance use and pain management theme at HMS, one of seven longitudinal themes in the undergraduate medical curriculum. Recognizing an opportunity to increase OUD education in the clerkship phase specifically, students on the SUP Curriculum Committee designed a peer-led, case-based workshop for Medicine Clerkship students which was focused on providing updated education on fentanyl, MOUD, acute pain management, and a harm reduction-informed approach to engaging hospitalized patients with OUD. We chose this content, in part, because we identified gaps in the current opioid education literature, which we ascertained using a keyword search of publications in the *MedEdPORTAL* Opioids, Addiction, and Pain Education collection since 2016. Our search revealed that among all patients reported to be taking opioids, 50% of published curricula mentioned fentanyl use, 46% mentioned MOUD use, and 58% mentioned naloxone use. Additionally, we chose to deliver this content using a near-peer teaching model, allowing senior students to deliver specialist-reviewed material while minimizing faculty time burden.[6-8] Finally, we integrated case-based learning, given its strong evidence base in medical education and its promotion of knowledge application through clinically relevant scenarios rather than passive reception.[9]

To our knowledge, this is the only workshop of its kind that utilizes near-peer teachers to deliver content tailored for students on their Medicine Clerkship. Although the Alliance for Academic Internal Medicine formally recommends including education on opioid intoxication, withdrawal, and OUD treatment in the Medicine Clerkship,[10] only half of all clerkships nationally cover topics related to safe opioid prescribing or OUD, signaling a continued need for updated curricula.[6] In our search, we identified one previously published opioid education intervention for students on their Medicine Clerkship involving a simulated patient interaction and a small-group case-based activity.[11] Notably, this didactic did not discuss acute pain management or MOUD pharmacology, was not peer-led, and did not assess student attitudes, knowledge, or confidence. The majority of other educational interventions involved opioid overdose reversal training and focused on assessing changes in students' knowledge, attitudes, and preparedness to respond to an overdose.[12-14] Other published curricula were formatted as transition-to-residency training for senior medical students or internship training for first-year residents; therefore, the emphasis was on withdrawal management and MOUD dosing but not counseling.[15-18] Additionally, none of these reviewed curricula included a discussion of fentanyl, possibly reflecting more recent shifts in the epidemiology of opioid overdose mortality.

Our intervention adds value by utilizing near-peer educators supervised by SUP experts and clerkship leaders, providing updated education about the role of fentanyl in the current overdose epidemic, including a discussion of acute pain management strategies for patients with OUD, and building translatable clinical skills using a real patient case with patient-provider counseling roleplay.

Methods

We designed, implemented, and evaluated a peer-led, case-based workshop for medical students on their Core Medicine Clerkship. To evaluate its effectiveness, we developed presession, postsession, and end-of-clerkship surveys to measure changes in participants' knowledge, attitudes, and confidence in caring for patients with OUD in the inpatient setting. The workshop was determined by the HMS Educational Scholarship Review Committee to not require Institutional Review Board approval (October 3, 2024).

Setting and Participants

HMS students who were on their Medicine Clerkship rotation at Beth Israel Deaconess Medical Center (BIDMC) in Boston, Massachusetts, from February 2024 through April 2025, participated in the workshop. This included six student cohorts, each comprising 4–14 students completing the 12-week clerkship. Of note, BIDMC is one of three primary clerkship sites affiliated with HMS. As part of the substance use and pain management theme in the preclerkship curriculum, students had previously received basic teaching related to substance use and pain, including lectures on opioid intoxication and withdrawal, a case discussion about a patient with OUD, a transition-to-clerkship lecture about the role of opioids in pain management, and a roleplay scenario using motivational interviewing.

Three postclerkship HMS students (William C. Oles, Erika Lynn-Green, Rory Vu Mather) delivered the content under the direct supervision of the BIDMC Medicine Clerkship leadership. William C. Oles and Erika Lynn-Green had previously completed

Copyright © 2026 Oles et al. This is an open-access publication distributed under the terms of the Creative Commons Attribution license.

the HMS Medical Education Longitudinal Elective, which provides formal education in teaching strategies and roleplay exercises. Students on the HMS SUP Curriculum Committee iteratively developed the materials over 6 months and received expert guidance on both background work and workshop design from faculty in addiction psychiatry (Hilary S. Connery, Patrick McGuire) and internal medicine (Julia Lindenberg, Ryan E. Nelson, Amy R. Weinstein). We refined the curriculum following a pilot with other medical students on the SUP Curriculum Committee.

Curriculum Design and Educational Strategies

The workshop consisted of a 25-minute didactic lecture followed by an interactive, 35-minute case-based portion that built upon the earlier content and included patient-provider roleplay (Appendix A). The workshop covered six key content areas in total, and peer teachers began each session by teaching the first four areas as a lecture, including (1) the epidemiology of the opioid epidemic, highlighting the role of fentanyl in recent years; (2) common medical consequences observed in persons who inject drugs (PWID); (3) principles of inpatient acute pain management for patients with OUD; and (4) the mechanisms of action of MOUD, comparing methadone, buprenorphine, naltrexone, and naloxone.

The final two content areas were integrated into a case (written by Erika Lynn-Green) based on representative experiences from an inpatient addiction psychiatry consult service and included (5) identifying questions to ask PWID during history-taking, including ways in which they engage in harm reduction; and (6) counseling on MOUD and inpatient harm reduction. The case follows a 25-year-old patient who injects fentanyl, presents to the hospital with a skin and soft tissue infection, and is eventually interested in learning about MOUD. In addition to identifying key questions during history-taking, students were asked to identify harm-reducing behaviors, describe important physical examination findings, and summarize their medical assessment of the patient's presentation. Finally, students reviewed the principles of motivational interviewing and discussed in pairs how they would approach a conversation with a patient interested in starting MOUD. The peer teacher then asked one or more student volunteers to engage in a roleplay with the peer teacher acting as the patient. Students received a postsession reference handout containing summaries on pertinent history questions, pain management for patients with OUD, MOUD, and overdose recognition and response (Appendix B). Experts (Hilary S. Connery, Patrick McGuire, Julia Lindenberg, Ryan E. Nelson, Amy R. Weinstein) reviewed the content to ensure concordance with local practices.

Implementation of the Curriculum

In total, we delivered workshops to six medical student clerkship cohorts in February 2024, May 2024, July 2024, October 2024, January 2025, and April 2025 as part of a preexisting, weekly, 1-hour didactic series for students in the Medicine Clerkship. Although attendance was required as part of the clerkship didactic series, completion of the associated surveys was optional, and no financial incentives were provided for participation. We used a conference room with a computer and a large screen monitor for the session. We developed a facilitator guide describing survey administration and content presentation to ensure that workshop content was delivered consistently and so that each session could be run by a different peer educator if needed (Appendix C).

Evaluation

We utilized a multiple-methods strategy to assess attitudes, knowledge, and confidence across a variety of domains aligned with the workshop's educational objectives (Appendix D). This approach follows the Kirkpatrick Model to provide a quantitative assessment of learning (level 2) via survey and a qualitative assessment of reactions to workshop delivery (level 1) and clinical behavior change (level 3) via a focus group.[19] We drafted presession, postsession, and end-of-clerkship surveys based on input from local experts in medical education (Ryan E. Nelson, Amy R. Weinstein), and administered the surveys as paper forms to maximize completion. We made final changes based on pilot testing with nonclerkship medical students to address response process validity.

To assess student attitudes about working with patients with OUD, we used the validated, 11-item Medical Condition Regard Scale (MCRS), adapted to include patients with OUD.[20] We assessed knowledge with a multiple-choice question, adapted from Fujita and colleagues, on managing acute pain in patients taking methadone therapy, and using the validated, 12-item Brief Opioid Overdose Knowledge (BOOK) scale.[17,21] We evaluated student confidence with two measures: a single question, adapted from Marshall and colleagues,[15] assessing confidence in knowledge of working with patients with OUD, and a second five-item survey on confidence related to workshop-specific educational objectives. In addition, on the end-of-clerkship survey, we asked students to record the number of patients with OUD they encountered. We excluded the acute pain multiple-choice question from the end-of-clerkship survey to reduce the survey length.

Finally, we invited all students who had attended the didactic to join a virtual focus group in May 2025. We developed guiding

Copyright © 2026 Oles et al. This is an open-access publication distributed under the terms of the Creative Commons Attribution license.

questions for the focus group to explore students' perceptions of the peer-led format and roleplay activity, to evaluate the session's impact on their clinical attitudes and practice, and to provide suggestions for improvement. The focus group was moderated via Zoom, lasted 1 hour, and was recorded with permission from the participants.

Data Analysis

We included all students who completed both the pre- and postsession surveys in a paired analysis. We compared the mean Likert-scale response for individual attitudes and confidence items, the mean MCRS cumulative score, the proportion of correct responses to individual knowledge items, and mean BOOK subscale scores. We checked all numericized Likert-scale responses against nonparametric paired sign tests to ensure consistency.[22] For students who also completed the end-of-clerkship survey, responses were analyzed using the Friedman test or Cochran's Q test. A p value less than .05 was considered statistically significant. R Statistical Software (v4.3.2; R Core Team 2023) was used to perform all quantitative analyses. The focus group was recorded, transcribed and anonymized, and then qualitatively analyzed by two collaborators (William C. Oles, Michael Motoc), who independently identified and finalized themes using content analysis.

Results

Sixty clerkship medical students attended one of the six workshops, and of these, 42 students at least partially completed the pre- and postsession surveys. Fifteen students fully completed the presession, postsession, and end-of-clerkship surveys. When weighted such that more desirable attitudes had higher scores, the mean (SD) cumulative MCRS score (out of a total score of 66) for student attitudes about working with patients with OUD was 48.8 (5.4) presession and 52.9 (5.4) postsession ($p < .001$). After the workshop, students were significantly more likely to indicate that working with patients with OUD is satisfying ($p < .001$), that they can find something to make these patients feel better ($p < .001$), and that insurance should cover patients with OUD to the same degree as other patients ($p = .003$; Table 1). In addition, after the workshop, students were significantly less likely to indicate that patients with OUD are difficult to work with ($p < .001$), that they prefer not to work with patients with OUD ($p = .03$), and that there is little they can do to help patients with OUD ($p = .03$).

In response to the multiple-choice question about acute pain management, there was a nonsignificant increase in the percentage of students who correctly identified the need for both long- and short-acting opioids (65% presession vs 77% postsession; $p = .26$). We noted a significant improvement in the proportion of students correctly identifying methadone as a long-acting opioid, as measured on the BOOK scale (69% presession vs 88% postsession; $p = .01$) and as the BOOK general opioid knowledge subscale score ($p = .004$). All respondents identified naloxone as an opioid overdose reversal agent, both before and after the workshop.

Finally, there were significant increases in all self-reported confidence measures, including feeling knowledgeable about working with patients with OUD ($p < .001$), describing common medical concerns of PWID ($p < .001$), describing acute pain management strategies for patients with OUD ($p < .001$), identifying examples of harm reduction in practice ($p < .001$), asking patients about opioid use ($p = .02$), and counseling patients about MOUD ($p < .001$) (Figure).

In subanalyses of students who completed the presession, postsession, and end-of-clerkship surveys, we noted that improvements in most MCRS items and all confidence items, but

Table 1. Medical Condition Regard Scale (Adapted) Scores of Student Attitudes Before and After the Workshop on Caring for Patients With Opioid Use Disorder ($N = 38$)

Survey Question[a]	Pre *M*	Post *M*	*p*
I prefer not to work with patients with opioid use disorder.	1.9	1.6	.03
Patients with opioid use disorder irritate me.	1.6	1.5	.13
I enjoy giving extra time to patients with opioid use disorder.	3.9	4.0	.38
Patients with opioid use disorder are particularly difficult for me to work with.	3.2	2.5	<.001
Working with patients with opioid use disorder is satisfying.	4.1	4.7	<.001
I feel especially compassionate toward patients with opioid use disorder.	4.3	4.5	.16
I wouldn't mind getting up on call nights to care for patients with opioid use disorder.	3.7	4.0	.15
I can usually find something that helps patients with opioid use disorder feel better.	3.4	4.2	<.001
There is little I can do to help patients with opioid use disorder.	2.2	1.8	.03
Insurance plans should cover patients with opioid use disorder to the same degree that they cover patients with other conditions.	5.2	5.4	.003
Treating patients with opioid use disorder is an ineffective use of medical dollars.	1.5	1.6	.60

[a]Rated on a 6-point Likert scale (1 = *strongly disagree*, 6 = *strongly agree*).

Copyright © 2026 Oles et al. This is an open-access publication distributed under the terms of the Creative Commons Attribution license.

MedEdPORTAL
THE AAMC JOURNAL OF TEACHING AND LEARNING RESOURCES

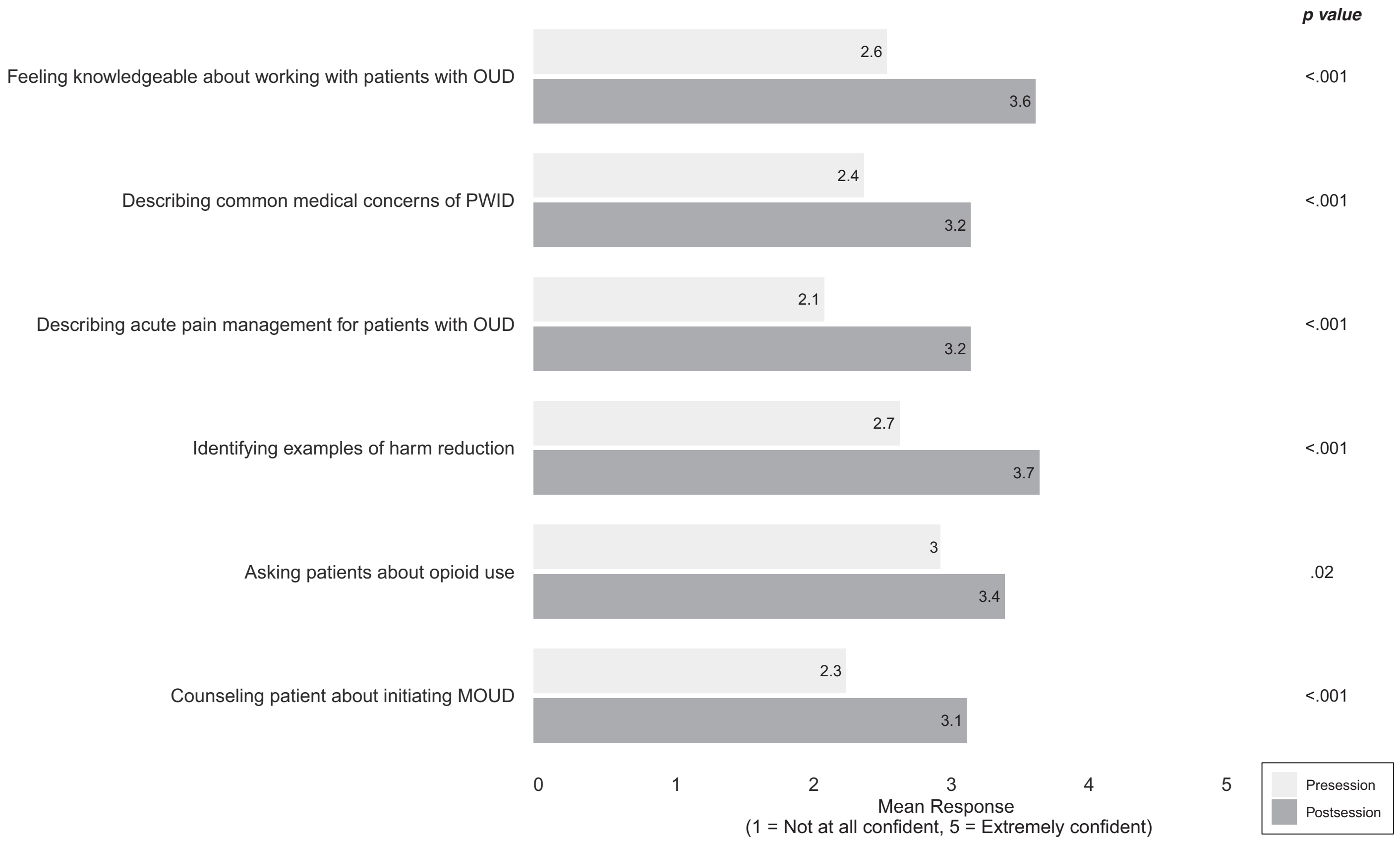

Figure. Student self-evaluated confidence levels immediately before and after the workshop on caring for patients with OUD. Mean confidence scores were rated on a 5-point Likert scale, with *p* values indicating significant differences between pre- and postsession responses. Abbreviations: MOUD, medications for opioid use disorder; OUD, opioid use disorder; PWID, persons who inject drugs.

not in knowledge about methadone, were sustained through the end of the clerkship. Students reported encountering an average of 3.5 patients with OUD during their clerkship experience.

Four students participated in the focus group hosted in May 2025. Themes identified in the focus group included (1) clinical applicability of the material; (2) importance of addressing stigma around OUD; (3) support for peer-led format; and (4) importance of accessible resources (Table 2). Participants described applying content about MOUD pharmacology and acute pain management to their practice and feeling more comfortable participating in conversations with patients. Students also emphasized the approachability of the peer-led format but cautioned against having a casual learning environment.

Regarding areas for future improvement, students shared a need for more clinical references to guide decision-making, the importance of hearing personal perspectives from patients and providers, and a concern that roleplay exercises can feel unproductive due to inadequate simulation of behavioral complexity experienced in real patient encounters.

Discussion

Given the ongoing opioid epidemic driven by illicitly manufactured fentanyl exposures and the relative dearth of published data on medical school curricula addressing opioid use and OUD, we developed a successful peer-led, case-based workshop for students on the Medicine Clerkship aimed at enhancing competency to care for patients with OUD. Overall, we noted a significant improvement in attitudes about working with patients with OUD, an increase in general knowledge about opioids, and an increase in confidence related to the session's educational objectives. However, these changes did not extend to all attitudes and knowledge assessed, and importantly, the translation of these changes to skill acquisition was not evaluated. Many, but not all, of the improvements noted after the teaching session persisted through the end of the clerkship;

Copyright © 2026 Oles et al. This is an open-access publication distributed under the terms of the Creative Commons Attribution license.

Table 2. Focus Group Themes and Representative Quotes

Theme	Quotes
Clinical applicability of material	"One thing that was really helpful was going back to the pharmacology… of buprenorphine and methadone. I remember talking to a patient about these medications [who] brought up fears that were mentioned in the didactic, and it was helpful to navigate that conversation."
	"I was grateful for the discussion about how to manage acute pain in a patient with opioid tolerance. That was something that I had never seen before, and what I learned in the lecture dovetailed nicely into what I learned in the rotation."
Importance of addressing stigma around opioid use disorder and opioid prescribing	"Going into [clerkship], I had a lot of fear around opioids because of things in the media. I think it's necessary to destigmatize."
	"I hadn't realized how intimidated a lot of practitioners are [to] prescribe these medications… and manage liability. I wasn't aware of how much hesitation there was until I started seeing it debunked."
	"I was uncomfortable addressing [these] issues when it came up with patients. So I think it made me less nervous when I was trying to have discussions and be involved in care."
Support for peer-led format	"I did like that it was led by a student. That made it feel less stressful and easier to ask questions."
	"I don't think it matters to me if it's a faculty member or student [teaching]. What matters is what sticks. Both students and faculty are credible. And it was awesome seeing a student share what they worked on."
	"I think there's a nice comfort with having a peer or near-peer teacher. It feels more approachable, especially for a topic where people might feel uncomfortable. The flip side is that sometimes it feels almost too casual and not driven home enough."
Importance of accessible resources	"Having an easy way to re-reference the framework in the future would be really helpful. The medicine rotation is so short, but we're going to be doing work in medicine for much longer."
	"What I really appreciated about the didactic is [that] it showed me how much it's part of the fabric of hospital training. It showed me that if I had questions, there are people I could go to, that there [are] resources available, and people are thinking about this."
	"Understanding hospital resources more broadly for people with substance use disorders would be helpful—knowing who to consult and when, and what resources are available at discharge."

however, this analysis was limited by survey response attrition. Both student feedback and the self-reported number of patients with OUD encountered supported the clinical applicability of the material. Our findings reinforce existing literature showing that opioid education targeted toward medical students is viewed positively, can influence attitudes toward this patient population, and can improve students' confidence in responding to patients with OUD.[11,15] However, our workshop also offered a unique opportunity to expand the educational content on overdose response beyond that emphasized by other, similar curricula to integrate an updated discussion of the role of fentanyl, address pain management in patients with OUD, and increase confidence in counseling about MOUD through patient-provider roleplay.

Several lessons emerged from reflections on the implementation of this educational intervention. Unlike previously published curricula, this workshop was led by near-peer educators, which increased learner comfort with asking questions during the session and allowed for unique clinical experiences to be shared by peer teachers. Peer teachers also found the experience rewarding, citing improved confidence in teaching skills and gratitude to past peer teachers as motivators for their involvement. The success is consistent with other peer-led models of medical education and extends the use of near-peer teaching to include OUD education.[7,8] We also found that student involvement helped address an outsized demand for faculty teaching, as clerkship faculty are motivated to deliver this content but are often limited by clinical and professional obligations not held by students. We believe the case discussion provided insight into the challenges many patients with OUD face, which can often be invisible to others, and was likely most contributory to the positive trends in student attitudes that we observed; however, this effect may be independent of the roleplay activity, which was found to be a less productive teaching method during the focus group. Finally, we also noted that by tailoring the workshop to skills that clerkship students can bring to the wards, the positive impact of this education had the potential to be 'taught up' to medical residents and other trainees on the students' inpatient care teams.

Our study had several limitations. This was a new workshop being trialed at a single institution and was therefore limited to small cohorts of students. This restricted the power to detect changes in students' knowledge, attitudes, or confidence about caring for patients with OUD that could be attributed to the educational content of the workshop, and limits the generalizability of our findings. A strength of our study was the use of validated scales in our survey instruments; however, some scales were adapted or were not directly aligned with our curriculum's content. Therefore, it is unclear whether our curriculum did not impact some attitudes and knowledge or whether relevant attitudes and knowledge were not assessed. Additionally, our survey revealed that participants had high baseline knowledge about opioid overdose recognition and response, signaling that limited instructional time could be prioritized for other content, such as MOUD. Finally, although

Copyright © 2026 Oles et al. This is an open-access publication distributed under the terms of the Creative Commons Attribution license.

efforts were made to make workshop content broadly applicable, the standards for some practices discussed may vary between institutions. We recommend that future presenters review the materials with clinician faculty in their program to ensure concordance with local practices, particularly regarding the availability and use of MOUD in the inpatient setting.

Given our finding that students have high baseline knowledge and attitudes in some domains, future work will benefit from including a control group of students to further validate our positive outcomes. The workshop itself may also be refined to include more patient and provider perspectives and to improve implementation of the roleplay activity. Future efforts will also benefit from refining the evaluation strategy to directly reflect workshop content, and expanding its delivery to a larger number of students. Similar workshops might also utilize the opportunity to compare peer-led and faculty-led models or to translate improvements in student knowledge, attitudes, and confidence into initiatives that directly impact patient care, such as naloxone distribution.[23] Finally, based on student feedback, similar interventions will benefit from distributing tailored educational materials (for example, Appendix C) to serve as a reference as students develop their practice both during their clerkship and beyond.

In summary, the Medicine Clerkship provided a feasible and unique opportunity to deliver multifaceted education about the care of patients with OUD in the hospital setting. While most undergraduate medical education emphasizes attaining medical knowledge, our educational intervention also sought to provide students with wards-relevant skills that we believe will enhance their ability to competently care for hospitalized patients with OUD moving forward.

Appendices

A. Presentation Materials.pptx

B. Postsession Handout.docx

C. Facilitator Guide.docx

D. Surveys.docx

All appendices are peer reviewed as integral parts of the Original Publication.

William C. Oles, MD: Resident Physician, Department of Medicine, University of California, San Francisco; ORCID: https://orcid.org/0000-0003-1125-6717

Erika Lynn-Green, MD, MSc: Resident Physician, Department of Medicine, Mayo Clinic

Rory Vu Mather, MS: Medical Student, Harvard Medical School

Michael Motoc, MA: Medical Student, Harvard Medical School

Allison Pellegrino: Medical Student, Harvard Medical School

Hilary S. Connery, MD, PhD: Assistant Professor of Psychiatry, Harvard Medical School; Clinical Director, Division of Alcohol, Drugs, and Addiction, Mass General Brigham McLean Hospital

Patrick McGuire, MD: Instructor in Psychiatry, Harvard Medical School; Medical Director, Addiction Recovery Program, Brigham and Women's Faulkner Hospital

Julia Lindenberg, MD: Assistant Professor of Medicine, Department of Medicine, Harvard Medical School and Beth Israel Deaconess Medical Center

Kevin P. Hill, MD, MHS: Associate Professor of Psychiatry, Division of Addiction Psychiatry, Harvard Medical School and Beth Israel Deaconess Medical Center

Stephen R. Pelletier, PhD: Assistant Director of Educational Scholarship and Faculty Development, Office of Educational Quality Improvement, Harvard Medical School

Ryan E. Nelson, MD: Assistant Professor of Medicine, Department of Medicine, Beth Israel Deaconess Medical Center; Associate Clerkship Director for the BIDMC Core I Medicine Clerkship, Harvard Medical School

Amy R. Weinstein, MD, MPH: Associate Professor of Medicine, Director of Student Programs, Department of Medicine, Beth Israel Deaconess Medical Center; Clerkship Director for the BIDMC Core I Medicine Clerkship, Harvard Medical School

Disclosures
None to report.

Funding/Support
None to report.

Ethical Approval
The Harvard Medical School Program in Medical Education's Educational Scholarship Review Committee reviewed this project.

References

1. Ahmad FB, Cisewski JA, Rossen LM, Sutton P. Provisional drug overdose death counts. National Center for Health Statistics. Accessed January 8, 2026. https://doi.org/10.15620/cdc/20250305008
2. Sordo L, Barrio G, Bravo MJ, et al. Mortality risk during and after opioid substitution treatment: systematic review and meta-analysis of cohort studies. *BMJ*. 2017;357:j1550. https://doi.org/10.1136/bmj.j1550

Copyright © 2026 Oles et al. This is an open-access publication distributed under the terms of the Creative Commons Attribution license.

3. Substance Abuse and Mental Health Services Administration. *Key substance use and mental health indicators in the United States: Results from the 2019 National Survey on Drug Use and Health*. HHS Publication No. PEP20-07-01-001, NSDUH Series H-55. Center for Behavioral Health Statistics and Quality, Substance Abuse and Mental Health Services Administration; 2020. https://www.samhsa.gov/data/sites/default/files/reports/rpt29393/2019NSDUHFFRPDFWHTML/2019NSDUHFFR090120.htm

4. McNeely J, Schatz D, Olfson M, Appleton N, Williams AR. How physician workforce shortages are hampering the response to the opioid crisis. *Psychiatr Serv*. 2022;73(5):547-554. https://doi.org/10.1176/appi.ps.202000565

5. Jo Y, Nosal R, Vittori A, et al. Effect of initiation of medications for opioid use disorder on hospitalization outcomes for endocarditis and osteomyelitis in a large private hospital system in the United States, 2014-18. *Addiction*. 2021;116(8):2127-2134. https://doi.org/10.1111/add.15393

6. Ari M, Kisielewski M, Osman NY, Szauter K, Packer CD, Pincavage AT. Teaching safe opioid prescribing during the opioid epidemic: Results of the 2018 Clerkship Directors in Internal Medicine survey. *J Gen Intern Med*. 2019;34(12):2812-2817. https://doi.org/10.1007/s11606-019-05203-3

7. Yu TC, Wilson NC, Singh PP, Lemanu DP, Hawken SJ, Hill AG. Medical students-as-teachers: a systematic review of peer-assisted teaching during medical school. *Adv Med Educ Pract*. 2011;2:157-172. https://doi.org/10.2147/AMEP.S14383

8. Rees EL, Quinn PJ, Davies B, Fotheringham V. How does peer teaching compare to faculty teaching? A systematic review and meta-analysis. *Med Teach*. 2016;38(8):829-837. https://doi.org/10.3109/0142159X.2015.1112888

9. McLean SF. Case-based learning and its application in medical and health-care fields: a review of worldwide literature. *J Med Educ Curric Dev*. 2016;3:JMECD-S20377. https://doi.org/10.4137/JMECD.S20377

10. Clerkship Directors in Internal Medicine (CDIM) Curriculum Task Force. *Core Medicine Clerkship Curriculum Guide*. 4th ed. Alliance for Academic Internal Medicine; 2020. https://uhmed.org/_docs/_clerkship/core-curriculum-guide.pdf

11. Tookes HE, Tomita-Barber J, Taldone S, et al. Opioid use disorder curriculum: medicine clerkship standardized patient case, small-group activity, and patient panel. *MedEdPORTAL*. 2022;18:11248. https://doi.org/10.15766/mep_2374-8265.11248

12. Sandhu RK, Heller MV, Buckanavage J, et al. A longitudinal study of naloxone opioid overdose awareness and reversal training for first-year medical students: specific elements require reinforcement. *Harm Reduct J*. 2022;19(1):70. https://doi.org/10.1186/s12954-022-00656-y

13. Oldfield BJ, Tetrault JM, Wilkins KM, Edelman EJ, Capurso NA. Opioid overdose prevention education for medical students: adopting harm reduction into mandatory clerkship curricula. *Subst Abus*. 2020;41(1):29-34. https://doi.org/10.1080/08897077.2019.1621241

14. Berland N, Lugassy D, Fox A, et al. Use of online opioid overdose prevention training for first year medical students: a comparative analysis of online vs in-person training. *Subst Abus*. 2019;40(2):240-246. https://doi.org/10.1080/08897077.2019.1572048

15. Marshall KF, Carney PA, Bonuck KJ, Riquelme P, Robbins J. Preparing fourth year medical students to care for patients with opioid use disorder: how this training affects their intention to seek addiction care opportunities during residency. *Med Educ Online*. 2023;28(1):2141602. https://doi.org/10.1080/10872981.2022.2141602

16. Estave PM, Jacobs ML, Rukstalis M, et al. Opioid stewardship training during the transition to residency to prepare medical students to recognize and treat opioid use disorder. *Subst Abus*. 2021;42(4):1040-1048. https://doi.org/10.1080/08897077.2021.1915918

17. Fujita AW, LaRosa A, Carter A. Treating withdrawal and pain in inpatients with opioid use disorder: a brief educational intervention for internal medicine residents. *MedEdPORTAL*. 2021;17:11123. https://doi.org/10.15766/mep_2374-8265.11123

18. Jennings L, Warner T, Bacro-Duverger B. Identification and treatment of opioid withdrawal and opioid use disorder in the emergency department. *MedEdPORTAL*. 2020;16:10899. https://doi.org/10.15766/mep_2374-8265.10899

19. Kirkpatrick JD, Kirkpatrick WK. *Kirkpatrick's Four Levels of Training Evaluation*. ATD Press; 2016.

20. Christison G, Haviland MG, Riggs ML. The medical condition regard scale: measuring reactions to diagnoses. *Acad Med*. 2002;77(3):257-262. https://doi.org/10.1097/00001888-200203000-00017

21. Dunn KE, Barrett FS, Yepez-Laubach C, et al. Brief Opioid Overdose Knowledge (BOOK): A questionnaire to assess overdose knowledge in individuals who use illicit or prescribed opioids. *J Addict Med*. 2016;10(5):314-323. https://doi.org/10.1097/ADM.0000000000000235

22. Roberson PK, Shema SJ, Mundfrom DJ, Holmes TM. Analysis of paired Likert data: how to evaluate change and preference questions. *Fam Med*. 1995;27(10):671-675.

23. Moore PQ, Cheema N, Follman S, et al. Medical student screening for naloxone eligibility in the emergency department: A value-added role to fight the opioid epidemic. *MedEdPORTAL*. 2021;17:11196. https://doi.org/10.15766/mep_2374-8265.11196

Received: September 2, 2025
Accepted: December 1, 2025
Published: January 27, 2026

Copyright © 2026 Oles et al. This is an open-access publication distributed under the terms of the Creative Commons Attribution license.
